# Blood-generating heart-forming organoids recapitulate co-development of the human haematopoietic system and the embryonic heart

**DOI:** 10.1038/s41556-024-01526-4

**Published:** 2024-10-08

**Authors:** Miriana Dardano, Felix Kleemiß, Maike Kosanke, Dorina Lang, Liam Wilson, Annika Franke, Jana Teske, Akshatha Shivaraj, Jeanne de la Roche, Martin Fischer, Lucas Lange, Axel Schambach, Lika Drakhlis, Robert Zweigerdt

**Affiliations:** 1 Leibniz Research Laboratories for Biotechnology and Artificial Organs (LEBAO), Department of Cardiothoracic, Transplantation and Vascular Surgery (HTTG), REBIRTH–Research Center for Translational Regenerative Medicine, Hannover Medical School, Hannover, Germany; 2 Research Core Unit Genomics (RCUG), Hannover Medical School, Hannover, Germany; 3 Institute of Experimental Hematology, REBIRTH–Research Center for Translational Regenerative Medicine, Hannover Medical School, Hannover, Germany; 4 Institute for Neurophysiology, Hannover Medical School, Hannover, Germany; 5 Division of Hematology/Oncology, Boston Children’s Hospital, Harvard Medical School, Boston, MA USA

**Keywords:** Haematopoiesis, Embryogenesis, Pluripotent stem cells

## Abstract

Despite the biomedical importance of haematopoietic stem cells and haematopoietic progenitor cells, their in vitro stabilization in a developmental context has not been achieved due to limited knowledge of signals and markers specifying the multiple haematopoietic waves as well as ethically restricted access to the human embryo. Thus, an in vitro approach resembling aspects of haematopoietic development in the context of neighbouring tissues is of interest. Our established human pluripotent stem cell-derived heart-forming organoids (HFOs) recapitulate aspects of heart, vasculature and foregut co-development. Modulating HFO differentiation, we here report the generation of blood-generating HFOs. While maintaining a functional ventricular-like heart anlagen, blood-generating HFOs comprise a mesenchyme-embedded haemogenic endothelial layer encompassing multiple haematopoietic derivatives and haematopoietic progenitor cells with erythro-myeloid and lymphoid potential, reflecting aspects of primitive and definitive haematopoiesis. The model enables the morphologically structured co-development of cardiac, endothelial and multipotent haematopoietic tissues equivalent to the intra-embryonic haematopoietic region in vivo, promoting research on haematopoiesis in vitro.

## Abstract (web summary)

Dardano et al. generate human pluripotent stem cell-derived cardiac organoids capable of undergoing endothelial-to-haematopoietic transition and producing haematopoietic cells.

## Main

Human pluripotent stem cells (hPSCs), including embryonic and induced (hESCs and hiPSCs, respectively) lines, can generate a broad range of organoids, that is, three-dimensional self-organized structures resembling tissue- and stage-specific aspects of embryogenesis in vitro^1,2^. Modelling tissue-integrated haematopoiesis in vitro is still limited yet fundamental for regenerative and developmental studies. Haematopoiesis is the process through which blood cells are generated; it occurs in three waves during human development^3–6^ derived from competent haemogenic endothelium (HE) via endothelial-to-haematopoietic transition^7,8^. The first two waves are haematopoietic stem cell (HSC)-independent and have mainly extra-embryonic origin at the level of the yolk sac. The first wave, termed ‘primitive haematopoiesis’, retains a limited erythro-myeloid potential and disappears during the fourth week of human development^9^. The second wave, termed ‘definitive transient haematopoiesis’, originates from erythro-myeloid and lympho-myeloid-primed progenitors that migrate from the yolk sac and intra-embryonic regions to the fetal liver to generate erythroid, myeloid and lymphoid derivatives^3,6,8,10,11^; some of these progenies will form long-lasting microglia and tissue-resident macrophages^12,13^, whereas most of the other derivatives will cease simultaneous to the nascent definitive HSC-dependent haematopoiesis. The latter, from which HSCs are generated, has an intra-embryonic origin that is the HE in the lumen of the dorsal aorta at the level of the aorta–gonad–mesonephros (AGM) region between four and six weeks of gestation^4,14,15^. Despite the biomedical importance of the haematopoietic system, its niche-like in vitro formation and stabilization in a proper developmental context remains elusive. Besides the inaccessibility of human embryos, underlying reasons include the complex and poorly understood mechanisms controlling haematopoietic development^8,11,16^. Addressing these challenges, definitive HE and haematopoietic progenitor cells (HPCs) have been successfully derived from hPSCs by the exogenous expression of transcription factors driving haematopoiesis^16,17^ or by the directed mesoderm priming of hPSC aggregates via WNT pathway activation and supplementation of growth factors such as bone morphogenetic protein 4 (BMP4), vascular endothelial growth factor (VEGF), basic fibroblast growth factor (bFGF) and stem cell factor (SCF)^18–24^. This promotes the therapeutically required production of haematopoietic derivatives^18,25^ without fully reflecting the multiple aspects of haematopoietic development. At present, only a few in vitro models present haematopoiesis together with relevant embryonic tissues, which is further limited to primitive haematopoiesis only^26,27^.

In a self-organized hPSC model termed heart-forming organoid (HFO), we recently showed the structured co-development of early heart, foregut and vascular anlagen^28,29^. We here report an advanced model named blood-generating (BG)-HFO. This model robustly resembles the co-development of heart anlagen (cardiomyocytes (CM) and septum transversum/pro-epicardial cells), arterial and venous endothelium, HE, HPCs and mature haematopoietic derivatives, reflecting different haematopoietic waves. The spatial cell/tissue location resembles the anatomy found in the embryo proper. Moreover, functional assays show the erythro-myeloid and lymphoid multipotency of BG-HFO-derived haemato-endothelial cells. The BG-HFO is thus a valuable model to study mechanisms of cardiac and haemato-endothelial development as well as specific properties of HPCs.

## Results

### BG-HFOs show functional myocardium and expanded endothelium

HFOs are generated by differentiating Matrigel-embedded hPSC aggregates via CHIR99021- (CHIR) and IWP2-mediated biphasic WNT pathway modulation^30,31^. The protocol induces an inner core of anterior foregut endoderm (AFE) and endothelial cells (ECs), an endocardial layer that separates the inner core from a myocardial layer (consisting of CMs), which is enclosed by an outer layer composed of CMs, septum transversum-like cells and posterior foregut endoderm (PFE); the whole structure is surrounded by mesenchymal cells (MES). Heart-forming organoids acquire a specific spatial multi-tissue organization, resembling anatomical aspects of cardiac and foregut development of the native embryo^28^.

**Fig. 1:**
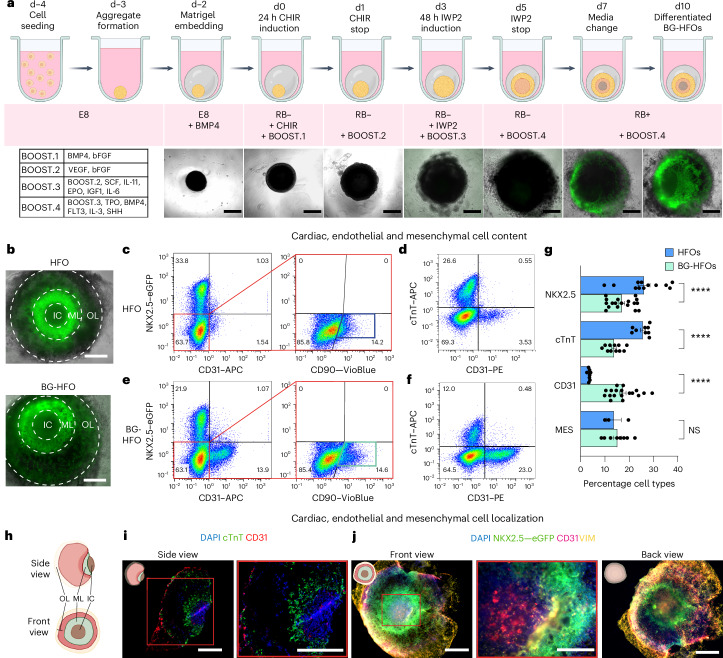
BG-HFOs display cardiac, endothelial and mesenchymal composition. **a**, Top: Protocol schematic for BG-HFO formation. Bottom left: Following Matrigel embedding, hPSC aggregates are differentiated using CHIR and IWP2 as well as specific time- and dose-dependent supplementation of haemato-endothelial-driving growth factors defined as BOOST.1–4. Bottom right: Overlay of light and fluorescence microscopy images depicting the time-dependent development of hES3 NKX2.5–eGFP-derived BG-HFOs from d−2 (embedding in Matrigel) until d10. **b**, Front view (see schematic in **h**) of a canonical hES3 NKX2.5–eGFP-derived HFO (top) and a BG-HFO (bottom), both forming three layers, that is, inner core, myocardial layer and outer layer. **c**,**e**, Representative flow cytometry plots of four or five homogenized HFOs (**c**) and BG-GFOs (**e**) to identify mesenchymal cells via the *NXK2.5–eGFP* transgene and staining for the endothelial cell marker CD31 as well as the fibroblast marker CD90. **d**,**f**, Representative flow cytometry plots of four or five homogenized HFOs (**d**) and BG-HFOs (**f**) gated on CD31 and the cardiac marker cTnT. **c**–**f**, The percentage of cells in the different quadrants is indicated. **g**, Proportions of cells expressing the markers NKX2.5–eGFP, cTnT and CD31 as well as mesenchymal cell content (NKX2.5–eGFP^−^CD31^−^CD90^+^ cells). Independent groups of four or five pooled HFOs (NKX2.5–eGFP, *n* = 12; cTnT, *n* = 9; CD31, *n* = 9; MES, *n* = 3) or BG-HFOs (NKX2.5–eGFP, *n* = 18; cTnT, *n* = 11; CD31, *n* = 19; MES, *n* = 8) from 14 independent experiments; data are presented as the mean ± s.e.m. One-way analysis of variance (ANOVA) with Bonferroni’s multiple comparison test; ****P < 0.0001; NS, not significant (*P* > 0.9999). **h**, Schematic showing the structure of a BG-HFO. **i**, Cryosections of a BG-HFO stained for cTnT and CD31 as well as with the nuclear marker 4,6-diamidino-2-phenylindole (DAPI). **j**, Whole-mount images of a BG-HFO immunofluorescently stained with DAPI, anti-VIM and anti-CD31. **i**,**j**, The pictograms (top left of images) depict the orientation of the organoids in the images. Right: Magnified views of the regions in the red boxes are shown. **a**,**b**,**i**,**j**, Scale bars, 500 µm. IC, inner core; ML, myocardial layer; and OL, outer layer.

To form BG-HFOs, the HFO protocol (schematic in Fig. 1a) was modified by supplementing growth factors and cytokines as previously established for hPSC differentiation to HPCs^19,21,32^. Specifically, pre-mesodermal priming by BMP4 on ‘day minus two’ (d−2) was followed by the addition of bFGF on d0, VEGF on d1 and a defined combination of additional factors from d3 onwards for haemato-endothelial differentiation in a stage-, factor- and dose-dependent manner (BOOST.1–4; Fig. 1a).

**Extended Data Fig. 1:**
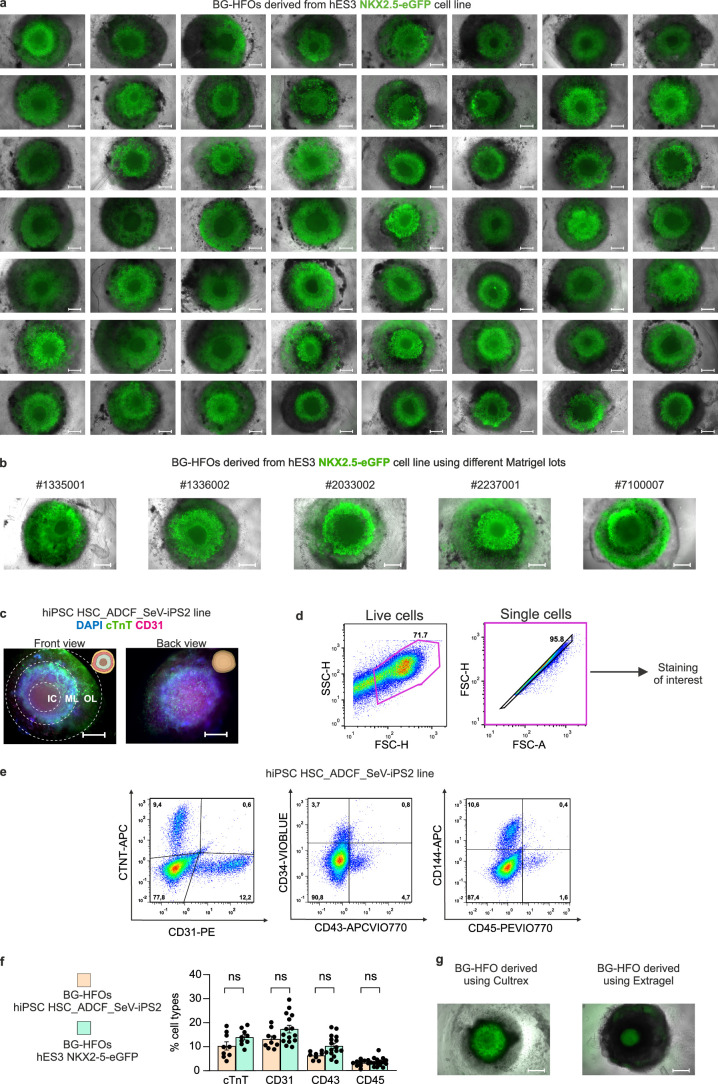
Robustness of the protocol. **a**. hES3 NKX2.5-eGFP-derived BG-HFOs from independent experiments showing robustness of the protocol. **b**. Representative images of 5 BG-HFOs obtained using 5 different lots of Matrigel. **c**. Whole-mount IF staining on a hiPSC HSC_ADCF_SeV-iPS220-derived BG-HFO, front and back, for DAPI, CD31 and cTnT, showing a classic cTnT ring-like pattern and localization of ECs in the IC and OL, equivalent to hES3 NKX2.5-eGFP-derived BG-HFOs; the pictograms on the IF staining represent the orientation of the organoid in the images. **d**. Representative gating strategy of the flow cytometry performed in this study. **e**. Representative flow cytometry plots of 4-5 homogenized hiPSC HSC_ADCF_SeV-iPS220-derived BG-HFOs for cTnT, CD31, CD144, CD34, CD43 and CD45. **f**. Percentages of cells expressing the markers cTnT, CD31, CD43 and CD45, independent groups of 4-5 pooled hiPSC HSC_ADCF_SeV-iPS220-derived BG-HFOs (cTnT n = 9; CD31 n = 9; CD43 n = 8; CD45 n = 10) from 5 independent experiments have been analysed and put in comparison to respective data from hES3 NKX2.5-eGFP-derived BG-HFOs (data shown in Fig. 1g); one-way ANOVA (Bonferroni’s multiple comparison test); data are presented as mean ± SEM; cTnT ns, P = 0.2221; CD31 ns, P = 0.0602; CD43 ns, P = 0.0956; CD45 ns, P > 0.9999. **g**. hES3 NKX2.5-eGFP-derived BG-HFOs in front view generated upon d-2 encapsulation in Cultrex or Extragel instead of Matrigel. Scale bars, (**a, b, c**) 500 µm.

The NKX2.5–eGFP hESC reporter line enables visualization of the typical HFO pattern (NKX2.5–eGFP^−^ inner core, NKX2.5–eGFP^+^ myocardial layer and partially NKX2.5–eGFP^+^ outer layer) from d7 onwards (Fig. 1a(bottom),b). Notably, BG-HFOs display a NKX2.5–eGFP pattern equivalent to HFOs in the front view on d10–14 (endpoint analysis; Fig. 1b,h). BG-HFO formation was highly robust between experiments as well as different Matrigel lots and was reproduced with an independent hPSC line (hiPSC HSC_ADCF_SeV-iPS220, ref. ^33^; Extended Data Fig. 1a–c).

Flow cytometry quantification of CMs (NKX2.5–eGFP^+^cardiac troponin-T (cTnT)^+^), ECs (CD31^+^) and MES (NKX2.5–eGFP^−^CD31^−^CD90^+^) revealed substantial differences in the composition of BG-HFOs versus HFOs (Fig. 1c–g and Extended Data Fig. 1d). The relative proportion of CMs was significantly reduced in BG-HFOs, whereas the EC content was about sixfold increased, representing almost 20% of cells in BG-HFOs; the MES content remained similar between the models (Fig. 1c–g). Equivalent results were obtained with HSC_ADCF_SeV-iPS220-derived BG-HFOs (Extended Data Fig. 1e,f). Alternative matrices (Cultrex and Extragel) resulted in failed BG-HFO formation, underpinning a Matrigel dependence (Extended Data Fig. 1g).

**Extended Data Fig. 2:**
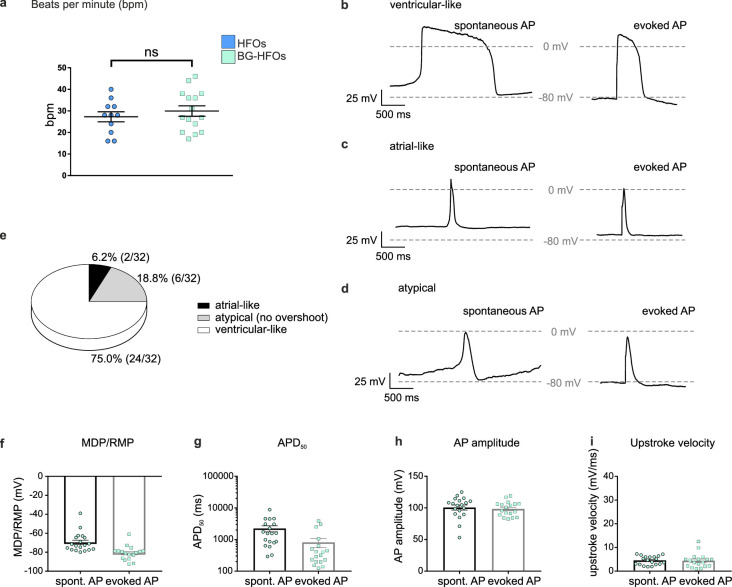
Functional and electrophysiological analysis of AP properties from BG-HFO-derived NKX2.5-eGFP^pos^ cardiomyocytes using whole-cell patch clamp technique in the current clamp mode. **a**. Beats per minute (bmp) measured in HFOs (n = 11) and BG-HFOs (n = 15) from 3 independent experiments, where each organoid is a independent biological unit; two-tailed, unpaired t-test assuming unequal variances for the comparison of two groups, data are presented as mean ± SEM; ns, P = 0.4468. **b**–**d**. Representative traces for action potentials (AP) from three different phenotypes of cardiomyocytes. Left panels show spontaneously initiated APs. Right panels depict evoked APs of the same cells, after hyperpolarizing the membrane to a comparable physiological resting potential around −80 mV by application of negative holding currents (−2 to −45 pA)^73^. In this case, APs were evoked by short depolarizing pulses (1 ms, 250–1,200 pA). Cells were classified as ventricular-like when APs showed a plateau phase longer than 200 ms as measured at 50% repolarization level (APD_50_) (**b**). Atrial-like cells displayed the typical triangular AP shape with APD_50_ values of 20–200 ms (**c**). Cells showing APs without overshoot were termed atypical (**d**). **e**. Distribution of BG-HFO-derived cell phenotypes based on the classification in **b**–**d**. **f**–**i**. Additional analysis of AP properties for ventricular-like cells isolated from BG-HFOs (**f**, MDP/RMP, maximal diastolic potential/resting membrane potential; **g**, APD_50_; **h**, AP amplitude; **i**, upstroke velocity). Spontaneous AP: n = 19; evoked AP: n = 18; data are presented as mean ± SEM.

Calcium imaging and fluorescence microscopy revealed distinct contraction patterns in BG-HFOs, that is, synchronous myocardial layer contraction (Supplementary Videos 1 and 2), circular beating (Supplementary Videos 3 and 4) and wave-like propagation (Supplementary Videos 5 and 6) equivalent to HFOs^28^. In addition, beats per minute analysis showed similar values between the two models (Extended Data Fig. 2a).

Equivalent to HFOs, patch clamping of BG-HFO-derived NKX2.5–eGFP^+^ CMs revealed some atypical action potential patterns and a minor population of atrial-like cells (6%); the majority (75%) of cells were classified as immature ventricular-like (Extended Data Fig. 2b–e) due to the low upstroke velocity (Extended Data Fig. 2f–i), consistent with previous work^28,30,34^. The maximal diastolic potential to resting membrane potential ratio, action potential duration at 50% of repolarization action potential amplitudes and upstroke velocities of ventricular-like cells were comparable between this organoid model and our previous study on HFOs (Extended Data Fig. 2f–i), suggesting similar CM phenotypes.

**Extended Data Fig. 3:**
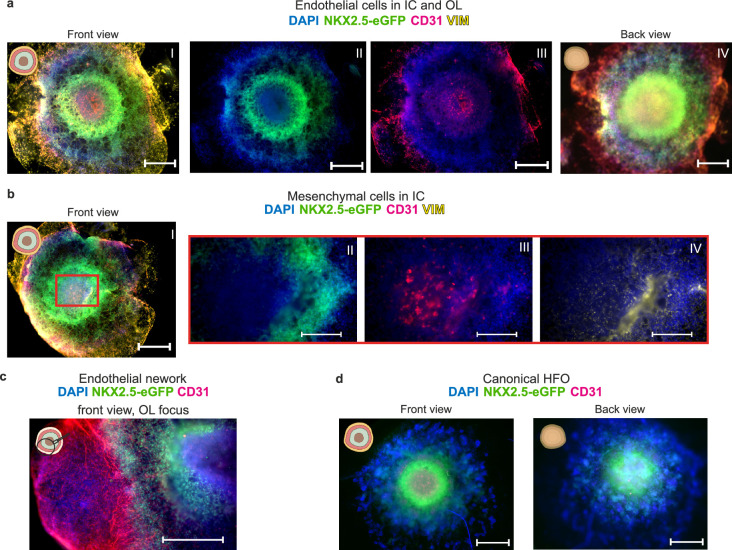
Endothelial, mesenchymal, and hematopoietic cell localization in BG-HFOs. **a**. Whole-mount IF staining on a BG-HFO for DAPI, vimentin (VIM) and CD31 (**I**), separated channel to visualize NKX2.5-eGFP^pos^ CMs (**II**), CD31^pos^ ECs (**III**), and the back ok the BG-HFO (**IV**), showing localization of ECs in the IC and OL. **b**. Whole-mount IF staining on a BG-HFO for DAPI, vimentin (VIM) and CD31 (**I**), magnification of the IC and separated channel to visualize NKX2.5-eGFP^pos^ CMs (**II**), CD31^pos^ ECs (**III**) and single-VIM^pos^ mesenchymal cells (**IV**), showing presence of mesenchyme in the IC. **c**. Whole-mount IF staining on a BG-HFO for DAPI and CD31 showing the endothelial network formed by the CD31^pos^ cells and pervading the organoid. **d**. Whole-mount IF staining on an HFO, front and back, for DAPI, and CD31, showing predominant localization of ECs in the IC, absence of ECs in the OL and presence of dense spherical structures in the OL representing PFE. The pictograms on the IF staining in **a**–**d** represent the orientation of the organoids in the images. Scale bars, (**a**–**d**) 500 µm.

Investigation of the proposed three-dimensional structure of BG-HFOs (Fig. 1h) in more detail by immunofluorescence analysis revealed that the inner core is enveloped by a cTnT^+^ myocardial layer and CD31^+^ ECs are present in the inner core (Fig. 1i), similar to HFOs^28^. Notably, although the outer layer of HFOs is mainly composed of CMs and PFE^28^, the outer layer of BG-HFOs contains mainly CMs and ECs but no PFE (Fig. 1i). Whole-mount immunofluorescence staining of BG-HFOs confirmed the presence of ECs in the inner core and the outer layer (Fig. 1j and Extended Data Fig. 3a). Similar to HFOs, vimentin-expressing (VIM^+^) MES were found in the outermost BG-HFO layer but only in the latter model were MES also found in the inner core (Fig. 1j and Extended Data Fig. 3b). No AFE was detected in BG-HFOs. Remarkably, in the outer layer of BG-HFOs, ECs form a dense branching endothelial network (Extended Data Fig. 3c).

Together, the HFO-typical AFE is replaced by ECs and MES in BG-HFOs. Moreover, whereas the outer layer of HFOs consists of PFE and CMs^28^, the endoderm is replaced by mesodermal derivatives, particularly ECs, in BG-HFOs (Extended Data Fig. 3a,d).

### BG-HFOs present endothelial subtypes and haematopoietic cells

Endothelium can be subdivided into vascular and HE^35^; vascular endothelium specifies into a venous (VE) or arterial (AE) subtype lining veins or arteries, respectively^4,8,36^, whereas HE is a heterogeneous population generating haematopoietic cells of the different waves^37–39^.

**Fig. 2:**
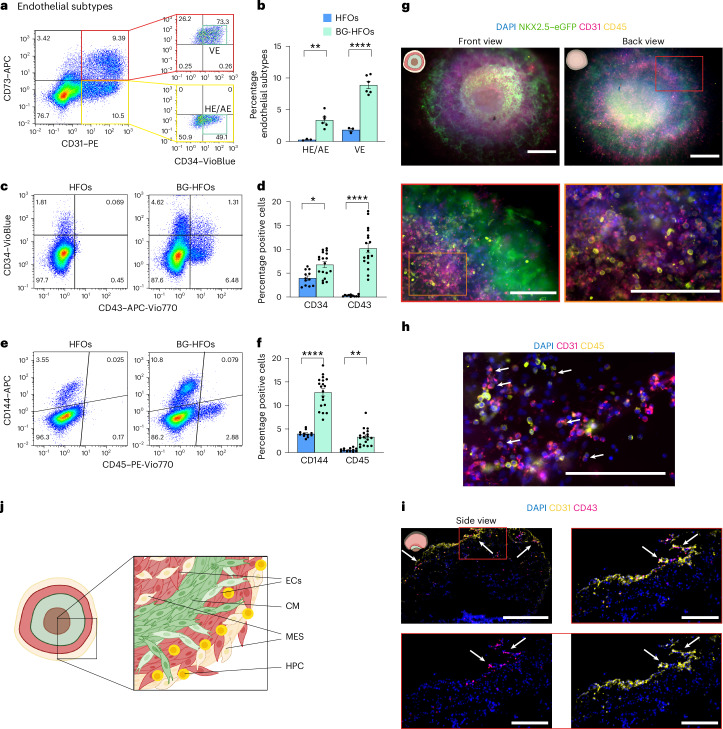
BG-HFOs display the presence of HE, VE, AE and HPCs. **a**, Representative flow cytometry plots of four or five homogenized BG-HFOs to identify the different endothelial subtypes, that is, AE/HE (CD73^−^CD31^+^CD34^+^) and VE (CD73^+^CD31^+^CD34^+^) endothelium. **b**, Proportion of AE/HE and VE in HFOs and BG-HFOs, determined using flow cytometry analysis. Independent groups of four or five pooled HFOs (AE/HE and VE, *n* = 3) and BG-HFOs (AE/HE and VE, *n* = 6) from four independent experiments were analysed; AE/HE, *P* = 0.0028. **c**,**e**, Representative flow cytometry plots of four or five homogenized HFOs and BG-HFOs to identify CD34^+^ endothelial and CD43^+^ haematopoietic cells (**c**) as well as CD144^+^ endothelial and CD45^+^ haematopoietic cells (**e**). **a**,**c**,**e**, The percentage of cells in the different quadrants is indicated. **d**,**f**, Proportion of CD34^+^ and CD43^+^ (**d**), and CD144^+^ and CD45^+^ (**f**) cells in HFOs and BG-HFOs determined from **c** and **e**, respectively. Independent groups of four or five pooled HFOs (CD34, CD43, CD144 and CD45, *n* = 12) and BG-HFOs (CD34 and CD43, *n* = 18; CD144 and CD45, *n* = 19) from 14 independent experiments were analysed. **d**, CD34, *P* = 0.0166. **f**, CD45, *P* = 0.0021. **g**, Outer layer of endothelial and haematopoietic cells. Front and back views of an hES3 NKX2.5–eGFP-derived BG-HFO following whole-mount immunofluorescence staining with DAPI, anti-CD31 and anti-CD45. Higher magnification images of the smaller red and orange boxes are outlined in the respective colours. **h**, Magnified view of the outer layer of the BG-HFO in **g** showing HPCs. **i**, Cryosection of a BG-HFO stained with DAPI, anti-CD31 and anti-CD43. Merged (top right) and separate channel (bottom) images of the region in the red box at higher magnification are provided. **h**,**i**, The arrows point to cells co-expressing CD31 and CD45, representing HPCs. **j**, Schematic of the cell composition of a BG-HFO. **b**,**d**,**f**, Data are presented as the mean ± s.e.m. One-way ANOVA with Bonferroni’s multiple comparison test; *P ≤ 0.05; **P ≤ 0.01; ****P ≤ 0.0001. **g**,**i**, The pictograms (top left in images) depict the orientation of the organoids in the images. **g**–**i**, Scale bars, 500 µm (main images in **g**,**i**) and 200 µm (**h** and magnified views in **g**,**i**).

**Extended Data Fig. 4:**
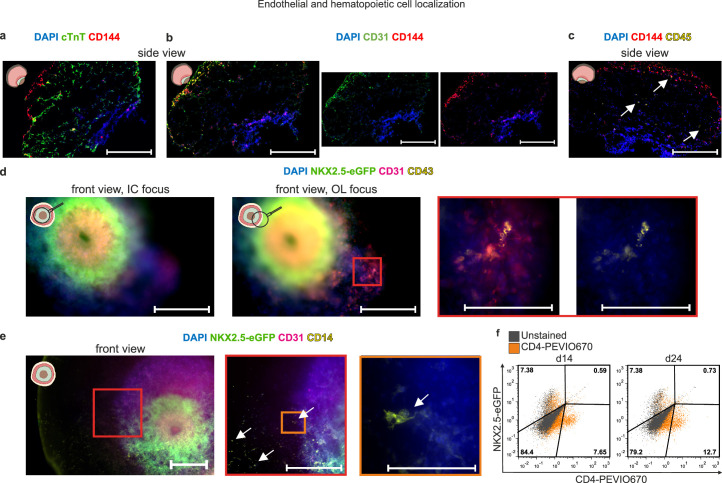
Endothelial and hematopoietic cell localization. **a–c.** Cryosections of a BG-HFO stained for DAPI, cTnT, and CD144 localizing the endothelial cells in the IC and OL (**a**), for DAPI, CD31, and CD144 localizing CD31^pos^/CD144^pos^ cells in both the IC and OL (separated channels to better visualize position of CD31^pos^ and CD144^pos^ cells) (**b**), and DAPI, CD144, and CD45, localizing endothelial cells and hematopoietic cells across the organoid (**c**). **d**. Whole-mount IF staining on a BG-HFO for DAPI, CD31, and CD43; focusing on the IC or the OL; magnification of the OL indicated by colour frames and separated channels to visualize areas of hematopoietic cells (CD43^pos^) and HPCs (CD31^pos^/CD43^pos^). **e**. Whole-mount IF staining on a BG-HFO for DAPI, CD31, and the myeloid marker CD14; consequential magnifications of the OL indicated by colour frames to visualize CD14^pos^ myeloid cells; some show cytoplasmatic extrusions typical of macrophages. **f**. Representative flow cytometry staining of a d14 and d24 BG-HFO for the myeloid/lymphoid marker CD4 (n = 2). The pictograms on the IF staining in **a**–**e** represent the orientation of the organoids in the images. Scale bars, (**a**–**d** first two images, **e** first image) 500 µm and, (**d** last images, **e** last images) 200 µm.

All ECs are characterized by the expression of CD31 and CD34, whereas CD73 is expressed at high levels preferentially, but not exclusively, in VE^36^. Flow cytometry revealed CD73^+^CD31^+^CD34^+^ cells in BG-HFOs, representing mostly VE (approximately 8% of all cells), and CD73^−^CD31^+^CD34^+^ cells, a heterogeneous population of putative AE and HE (approximately 4%; Fig. 2a)^36,37^. Only VE-like endothelium was observed in HFOs (Fig. 2b). Moreover, BG-HFOs showed significantly more CD144^+^ and CD34^+^ ECs (Fig. 2c–f), consistent with elevated CD31 expression (Fig. 1g). Immunofluorescence staining for CD144 and cTnT corroborated the localization of ECs in the inner core and outer layer of BG-HFOs, and CD144^+^ cells co-expressed CD31, as expected (Extended Data Fig. 4a,b).

In contrast to HFOs, BG-HFOs contain approximately 10% CD43^+^ (Fig. 2c,d) and 4% CD45^+^ cells (Fig. 2e,f), typical markers of HPCs and mature haematopoietic derivatives.

Using immunofluorescence staining, round CD45^+^ haematopoietic cells and CD45^+^CD31^+^ HPCs were localized in direct proximity to CD31^+^ or CD144^+^ endothelium in the outer layer (Fig. 2g,h and Extended Data Fig. 4c); cryosections and whole-mount immunofluorescence staining further corroborated the presence of round CD43^+^ haematopoietic cells and CD43^+^CD31^+^ HPCs mainly in the outer layer of BG-HFOs (Fig. 2i and Extended Data Fig. 4d).

Notably, monocytes and macrophages (CD14^+^)—some of which showed typical cytoplasmic extroflections of myeloid cells—were detected in the outer layer and the surrounding Matrigel (Extended Data Fig. 4e). Cells positive for the myeloid/lymphoid marker CD4 were also identified in d14 and d24 BG-HFOs using flow cytometry (Extended Data Fig. 4f).

**Extended Data Fig. 5:**
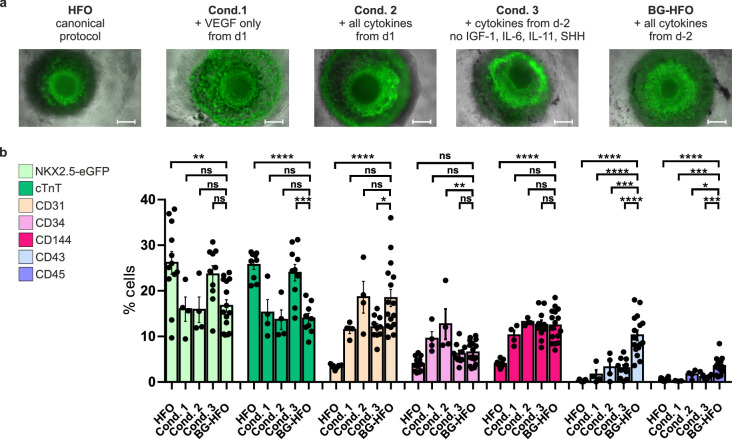
Depletion of molecules leads to impaired hematopoiesis in BG-HFOs. **a**. Front view of a hES3 NKX2.5-eGFP-derived HFO, Cond.1 organoid, Cond.2 organoid, Cond.3 organoid, and BG-HFO, all showing the typical IC-ML-OL pattern. **b**. Percentages of cells expressing the markers NKX2.5-eGFP, cTnT, CD31, CD34, CD144, CD43, and CD45; independent groups of 4–5 pooled HFOs (NKX2.5-eGFP, CD34, CD144, CD43, CD45 n = 12; cTnT, CD31 n = 9), BG-HFOs (NKX2.5-eGFP n = 15; cTnT n = 9; CD31, CD34, CD144, CD43, CD45 n = 16), Cond.1 (NKX2.5-eGFP, cTnT, CD31, CD34, CD144, CD43, CD45 n = 4), Cond.2 (NKX2.5-eGFP, cTnT, CD31, CD34, CD144, CD43, CD45 n = 4) or Cond.3 (NKX2.5-eGFP, cTnT, CD31, CD34, CD144, CD43, CD45 n = 10) organoids from in total 17 independent experiments have been analysed, one-way ANOVA (Sidak’s multiple comparison test); data are presented as mean ± SEM; for NKX2.5-eGFP: HFO/BG-HFO **P = 0.0024; Cond.1/BG-HFO ns, P =0.9994; Cond.2/BG-HFO ns, P = 0.9988; Cond.3/BG-HFO ns, P = 0.0527; for cTnT: HFO/BG-HFO ****P < 0.0001; Cond.1/BG-HFO ns, P = 0.9810; Cond.2/BG-HFO ns, P > 0.9999; Cond.3/BG-HFO ***P = 0.0001; for CD31: HFO/BG-HFO ****P <0.0001; Cond.1/BG-HFO ns, P = 0.0981; Cond.2/BG-HFO ns, P > 0.9999; Cond.3/BG-HFO *P = 0.0208; for CD34: HFO/BG-HFO ns, P = 0.0868; Cond.1/BG-HFO ns, P = 0.2288; Cond.2/BG-HFO **P = 0.0013; Cond.3/BG-HFO ns, P = 0.9984; for CD144: HFO/BG-HFO ****P < 0.0001; Cond.1/BG-HFO ns, P = 0.5186; Cond.2/BG-HFO ns, P = 0.9869; Cond.3/BG-HFO ns, P = 0.9999; for CD43: HFO/BG-HFO ****P < 0.0001; Cond.1/BG-HFO ****P < 0.0001; Cond.2/BG-HFO ***P = 0.0006; Cond.3/BG-HFO ****P < 0.0001; for CD45: HFO/BG-HFO ****P < 0.0001; Cond.1/BG-HFO ***P = 0.0004; Cond.2/BG-HFO *P = 0.0373; Cond.3/BG-HFO ***P = 0.0001. Scale bars, 500 μm.

The importance of supplemented factors (BMP4 and bFGF) at the early stages of differentiation (d−2 and d0) and the impact of some factors in the BOOST.3 and BOOST.4 mixture was investigated. Specifically, we tested the following: VEGF addition only (Cond.1), depletion of BMP4 and bFGF on d−2 and d0 (Cond.2) and removal of specific factors (Cond.3) based on published haematopoietic protocols^18,19,21,23,24,40,41^ (Extended Data Fig. 5a). Despite minor changes in the CM and EC content between Cond.1, Cond.2 and Cond.3 compared with BG-HFOs, the haematopoietic cell content (CD43^+^ and CD45^+^) was significantly lower in all modified conditions (Extended Data Fig. 5b).

**Extended Data Fig. 6:**
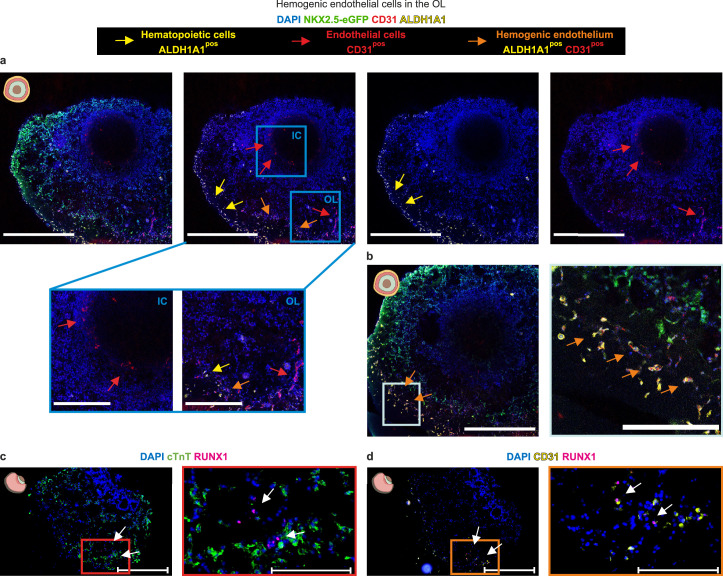
Haemogenic endothelium is specifically localized in the OL of BG-HFOs. **a**,**b**. Confocal microscopy on a whole-mount IF-stained BG-HFO. Images are from 2 different confocal plains of the same BG-HFO and show cardiomyocytes (NKX2.5-eGFP^pos^), hematopoietic cells (ALDH1A1^pos^) localized in the OL, endothelial cells (CD31^pos^) localized in both the OL and IC, and haemogenic endothelial cells (ALDH1A1^pos^/CD31^pos^) localized in the OL only; different cells are indicated by colour-coded arrows. Higher magnification images are indicated by colour frames. **c**,**d**. Consequential cryosections of a BG-HFO stained for DAPI, cTnT, and RUNX1 (**c**) and for DAPI, CD31, and RUNX1 (**d**), arrows indicate RUNX1^pos^/cTnT^neg^ cells (**c**); the same cells are RUNX1^pos^/CD31^pos^ (**d**), representing haemogenic endothelium in the OL. Higher magnification images are indicated by colour frames. The pictograms on the IF staining in **a**–**d** represent the orientation of the organoids in the images. Scale bars, (**a** upper images, **b** first image, **c** first image, **d** first image) 500 μm, (**a** lower images IC and OL, **b** second image, **c** second image, **d** second image) 200 μm.

To better localize the HE in BG-HFOs, we performed immunofluorescence staining of the transcription factor aldehyde dehydrogenase 1 family member A1 (ALDH1A1)—expressed in HE and emerging HSCs in the embryonic AGM region^24,42^—and found ALDH1A1 and CD31 co-expression indicative of HE predominantly in the outer layer (Extended Data Fig. 6a,b). Immunofluorescence staining of runt-related transcription factor 1 (RUNX1)—another established transcription factor in HE and HSC/HPC development—revealed clusters of positive cells in the outer layer (Extended Data Fig. 6c); on consecutive cryosections the RUNX1^+^ cluster co-expressed CD31, further corroborating the localization of HE in the outer layer (Extended Data Fig. 6d). Neither ALDH1A1^+^CD31^+^ nor RUNX1^+^CD31^+^ cells were detected in the inner core of BG-HFOs; only cells single-positive for CD31 representing other EC types, presumably VE, were detected.

Together, in contrast to HFOs, BG-HFOs include different subtypes of endothelium—that is, AE and HE (AE/HE), and VE—as well as HPCs. Whereas the CD31^+^ and CD144^+^ ECs pervade the whole organoid, the presence of ALDH1A1^+^CD31^+^ and RUNX1^+^CD31^+^ HE as well as CD45^+^ and CD43^+^ cells is exclusive to the outer layer and thus in a morphologically distinct area of BG-HFOs (Fig. 2j).

### Confirmation of various tissues in BG-HFOs via scRNA-seq

**Fig. 3:**
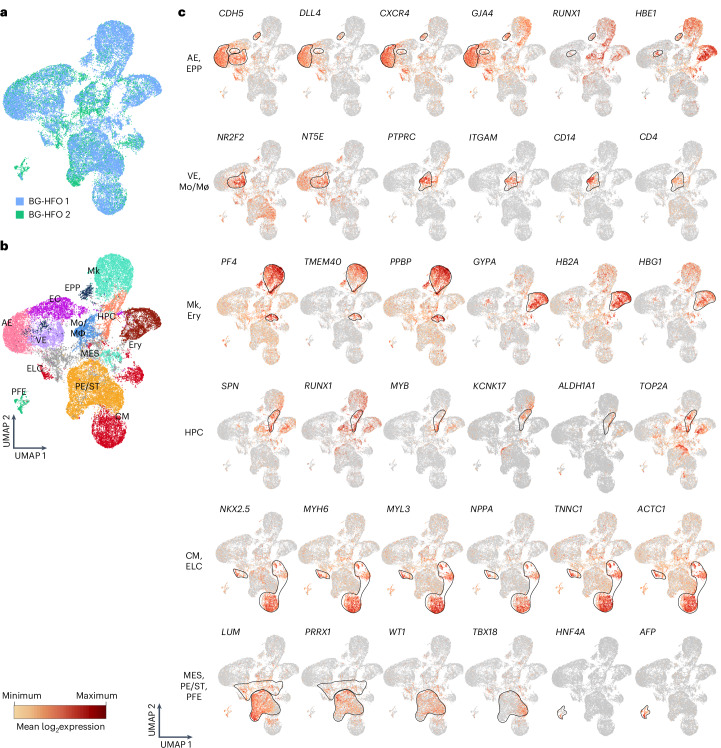
Presence of different endothelial subtypes, CMs and haematopoietic derivatives revealed by scRNA-seq. **a**, Uniform manifold approximation and projection (UMAP) plot of cells from two d14 BG-HFO samples colour-coded according to sample identity (10,000 cells for each sample). **b**, UMAP plot with cells colour-coded according to the graph-based clustering. Clusters were annotated based on differential expression of key genes. **c**, Feature plot of selected genes across all BG-HFO clusters are shown in the composite UMAP plots. The red colour gradient represents the level of expression of the indicated key genes. Regions outlined in black define the different clusters based on key genes expression.

Single-cell RNA sequencing (scRNA-seq) of two BG-HFOs showed equally positioned cell populations demonstrating robustness of tissue formation in individual organoids (Fig. 3a). Merging of sample data and clustering using Seurat functions and the Leiden algorithm revealed nineteen clusters, some of which were unified based on gene expression similarities to form thirteen major clusters (Fig. 3b). Each cluster was classified based on the expression of marker genes, confirming lineages suggested by immunofluorescence staining and flow cytometry.

**Fig. 4:**
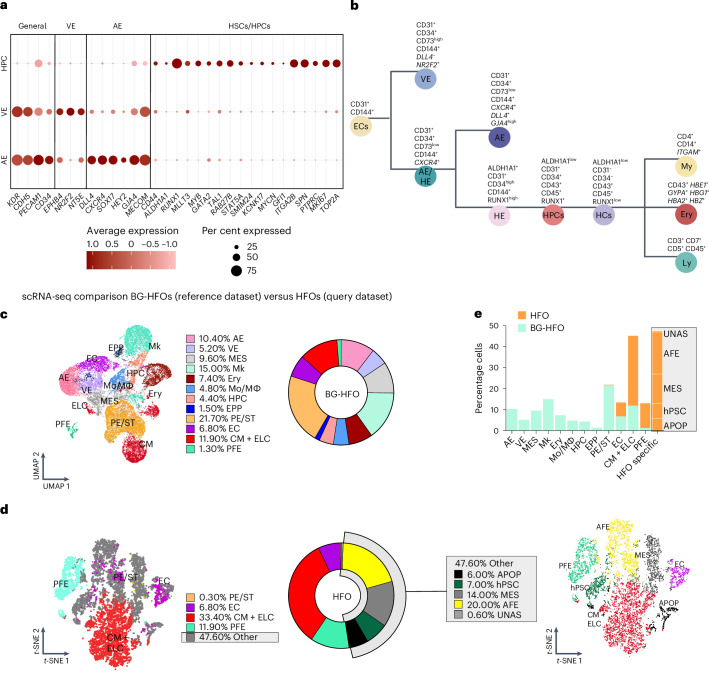
Identification of haemato-endothelial populations in BG-HFOs and a comparison between HFOs and BG-HFOs using scRNA-seq. **a**, Haemato-endothelial dot plot showing genes that are differentially expressed between the AE, VE and HSCs/HPCs. The dimension of the dots represents the percentage of cells expressing the specific gene, and the red colour gradient represents the level of expression. **b**, Schematic of the haemato-endothelial markers representing the multiple haemato-endothelial populations present in the BG-HFOs and markers used to identify them at gene (scRNA-seq) and protein expression (immunofluorescence staining, flow cytometry on BG-HFOs and functional analyses outcomes) levels. HCs, haematopoietic cells; My, myeloid derivatives; Ly, lymphoid derivatives. **c**, BG-HFO UMAP plot (reference dataset) and pie diagram with cells colour-coded according to the annotated cell types. Clusters were annotated based on differential expression of key genes. The names and cell percentages of each cluster are reported. **d**, *T*-Distributed stochastic neighbour embedding (*t*-SNE) plot and pie diagram of the HFOs query dataset^28^ with transferred cell-type annotation from the reference dataset (BG-HFOs) and original cell-type annotation from previous work^28^ (grey shaded). The names and cell percentages of each cluster are reported. APOP, apoptotic cells, UNAS, unassigned cells. **e**, Compositional analysis of HFOs and BG-HFOs. Cell-type identification based on expression of marker genes (BG-HFOs) and reference mapping (HFOs). Subsets of HFO-specific cell types were identified based on previous work^28^.

Cluster-specific expression of key lineage markers is highlighted in Fig. 3c. Three endothelial clusters were identified, all of which express the general EC genes, such as *CDH5* (CD144) and *PECAM1* (CD31), together representing approximately 25% of all cells. Two major endothelial clusters were characterized by the expression of AE and VE genes, comprising approximately 16% of all cells. The VE cluster displayed higher expression of the venous genes *NR2F2* (COUP-TFII) and *NT5E* (CD73), whereas the AE cluster was specified by the arterial markers *DLL4*, *CXCR4*, *GJA4*, *SOX17* and *MECOM*^15,42^ (Figs. 3c and 4a).

**Extended Data Fig. 7:**
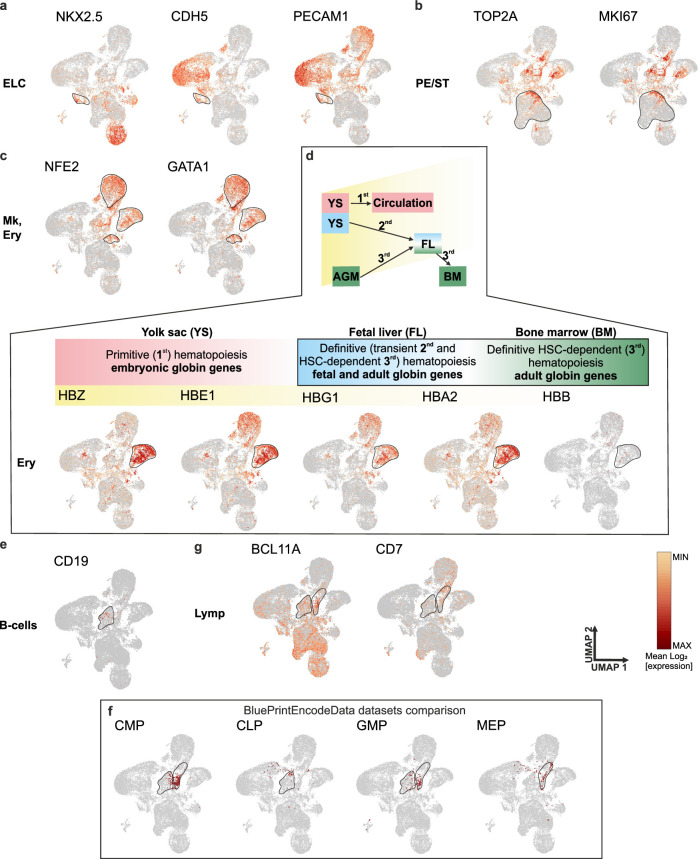
Expression of additional genes in BG-HFOs. **a**–**g**. Feature plot of indicated genes across all BG-HFO clusters (2 combined d14 BG-HFOs) shown in the composite UMAP plot, indicating co-expression of cardiac and endothelial genes in the ELC cluster (**a**), expression of proliferative genes in a part of the ST/PE cluster (**b**), co-expression of genes in Mk and Ery clusters (**c**), globin gene expression in the Ery cluster (**d**), indicating an embryo-fetal globin expression of erythroid derivatives forming the Ery cluster (**d**, representative scheme on top), expression of a B cell marker in the Mo/Mø cluster (**e**), Annotation generated comparing BG-HFOs’ scRNA-seq dataset to the BluePrintEncodeData datasets, revealing the presence of common myeloid progenitors (CMP), common lymphoid progenitors (CLP), granulocytes macrophages progenitors (GMP), and megakaryocytes erythroid progenitors (MEP) in BG-HFOs (**f**), and early lymphoid genes in the HPC cluster (**g**).

Cardiac-related clusters included CMs (approximately 11%), endocardial-like cells (ELCs; approximately 1%) and proepicardium/septum transversum (PE/ST; approximately 19%). Cardiomyocytes and ELC cells were characterized by the common expression of *NKX2.5*, *MYH6*, *MYL3*, *NPPA*, *TNNC1* and *ACTC1*; ELCs displayed additional expression of endothelial genes including *CDH5* and *PECAM1* (ref. ^43^; Fig. 3c and Extended Data Fig. 7a). The mesenchyme-derived PE/ST cluster was identified by the expression of *WT1*, *TBX18* and mesenchymal markers; part of this cluster expresses *TOP2A* and *MKI67*, indicating proliferation. A cluster expressing prevalently mesenchymal genes (MES; approximately 10% of all cells), including *LUM* and *PRRX1*, was also observed (Fig. 3c and Extended Data Fig. 7b).

Only a minor cluster expressing the PFE-related genes *AFP* and *HNF4A* was observed (approximately 1%)^28,44^ and no AFE was detectable, confirming the almost entire loss of endodermal tissue in BG-HFOs.

Four major haematopoiesis-related clusters were identified. A cluster of megakaryocytes (Mk, 13%) expressing *PF4*, *TMEM40* and *PPBP* was detected. Megakaryocytes are developmentally close to erythroid derivatives; the erythroid cluster (Ery, 7%) indeed shared expression of some genes with the megakaryocyte cluster (*NFE2* and *GATA1*; Extended Data Fig. 7c). The erythroid cluster, however, could be distinguished by high expression levels of *GYPA*, the fetal globin genes *HB2A* and *HBG1* as well as the embryonic globin genes *HBZ* and *HBE1* (Fig. 3c and Extended Data Fig. 7d).

A cluster of myeloid cells represented by monocytes/macrophages (Mo/Mø; 5%) was identified by the expression of early monocyte-related genes such as *PTPRC*, *ITGAM* and *CD14*, and macrophage-related maturation genes including *HLA-DRA*, *C1QA* and *CD4* (Fig. 3c). Notably, in the latter cluster, expression in a few cells of the B cell marker *CD19* indicates the putative presence of cells with lymphoid potential (Extended Data Fig. 7e).

Identification of HPCs based on gene expression patterns is challenging due to ambiguous markers and the multitude of simultaneous intermediates known to exist along the native haematopoietic differentiation^4,8,11^. However, it was possible to identify an HPC cluster in BG-HFOs (4%) by co-expression of factors related to HSC/HPC development (including *SPN* (CD43), *PTPRC* (CD45), *CD44*, *MYB*, *RUNX1*, *GATA2* and *TAL1*^15,19,42,45^ as well as the proliferation-associated markers *MKI67* and *TOP2A*), potentially reflecting the highly proliferative phenotype of HPCs in vivo. Genes recently identified by Calvanese and colleagues^42^ to be enriched in HSCs (*ALDH1A1*, *MLLT3*, *MYCN*, *GFI1*, *KCNK17*, *RAB27B* and *ITGA2B*) were, to some degree, also expressed in the HPC cluster, whereas genes such as *HOXA9*, *MECOM*, *HLF* and *SPINK2* were missing, indicating an absence of proper HSCs^42^ (Figs. 3c and 4a).

Annotation of our results in the ‘BlueprintEncodeData’ database confirmed the presence of common myeloid progenitors, granulocyte–macrophage progenitors and megakaryocyte–erythroid progenitors in the HPC cluster; in addition, a few cells annotated as common lymphoid progenitors were detected in the Mo/Mø cluster (Extended Data Fig. 7f).

Importantly, we detected some *BCL11A-* and *CD7-*expressing cells in both the Mo/Mø and HPC clusters, which suggests the presence of early progenitors with putative lymphoid potential (Extended Data Fig. 7g).

A minor cluster co-expressing the mentioned AE genes, the HE/haematopoietic-related genes *RUNX1* and *SPN*, and embryonic-fetal globin genes may represent very immature haematopoietic cells^46^, potential early primitive progenitors (EPP; 2%, Fig. 3c).

Together, the scRNA-seq analysis confirmed derivatives of heart anlagen (CMs and ELCs), derivatives of the mesenchyme (PE/ST and MES) and endothelial subtypes (AE and VE) in BG-HFOs, revealed more details on haematopoietic derivatives—including HPCs and erythro-myeloid cells (megakaryocytes, erythroids and Mo\Mø)—and indicated the presence of cells expressing early lymphoid progenitor genes.

Haemato-endothelial-related markers observed by scRNA-seq, immunofluorescence staining, flow cytometry and functional assays were collected in a lineage tree in Fig. 4b.

Comparison of scRNA-seq data of BG-HFOs and HFOs^28^ further elucidated analogies and differences between both models. The comparison confirmed high similarities in the gene expression signature of the CM clusters (Fig. 4c–e), corroborating both the flow cytometry analysis (Fig. 1c–g) and the functional assessment (Extended Data Fig. 2a–i). Notably, the PE/ST cluster is expanded in BG-HFOs compared with HFOs, in contrast to the reduced amount of CMs.

The MES cluster of HFOs and BG-HFOs presented a similar expression of key mesenchymal genes such as *PRRX1*, *LUM* and *TWIST1*, although the overall expression patterns were not similar enough for the annotation, possibly reflecting different mesenchymal subtypes (Fig. 4c–e). Furthermore, the gene expression signature of the minor PFE cluster in BG-HFOs was comparable to the larger PFE cluster in HFOs.

The EC cluster in HFOs displayed gene expression similarities to the EC cluster in BG-HFOs but not the AE cluster (Fig. 4c–e). As expected, neither the AE nor the haematopoietic clusters (Mk, Ery, Mo/Mø, EPPs and HPCs) were detected in HFOs; vice versa, the AFE and hPSC clusters in HFOs^28^ were not detectable in BG-HFOs (Fig. 4c–e).

**Extended Data Fig. 8:**
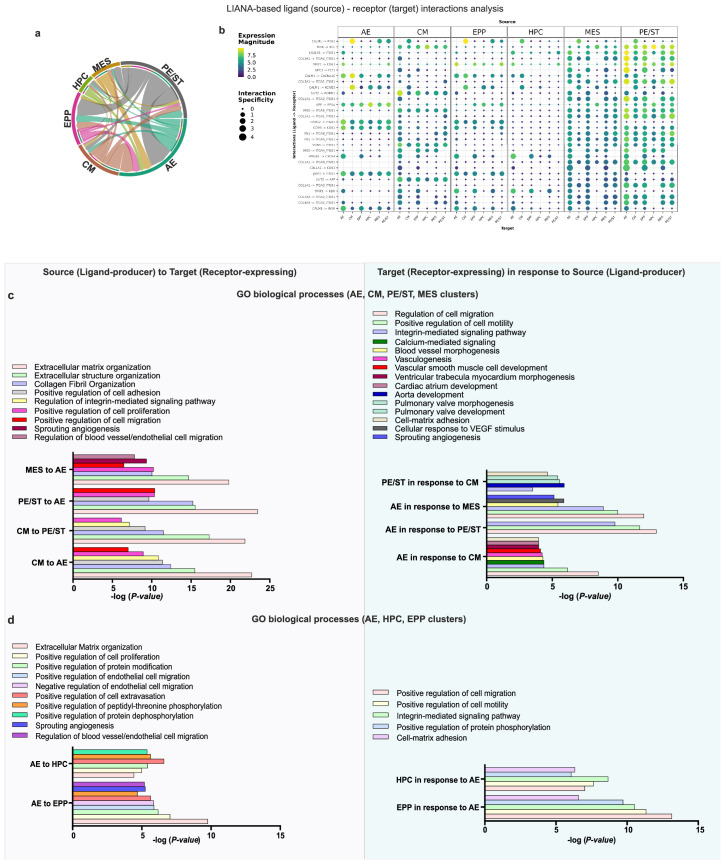
scRNA-seq-based ligand–receptor interaction analyses. **a**. Frequency chord diagram displaying the interaction frequencies of chosen sources and target cell type groups in BG-HFOs (CM, AE, PE/ST, MES, HPC, EPP) **b**. Dot plot displaying the specific ligand–receptor interactions between source (CM, AE, PE/ST, MES, HPC, EPP) to target (CM, AE, PE/ST, MES, HPC, EPP) cells. ‘Specificity’ refers to how specific this interaction is to a cell type pair relative to the rest of the cell type pairs, while ‘Magnitude’ is a direct measure of the expression. The 30 interactions with the highest ‘Magnitude’ and ‘Specificity’ are shown. **c**,**d**. Gene set enrichment analysis using the Enrichr and the GO biological process 2023 database for detected ligands and receptors (magnitude rank < 0.01) in CM, AE, PE/ST, and MES (**c**) or AE, HPC and EPP (**d**) obtained via LIANA. The used p-values are computed from the Fisher exact test which is a proportion test that assumes a binomial distribution and independence for probability of any gene belonging to any set.

For insights into cell–cell communication (CCC), which is pivotal in tissue development, a ligand–receptor analysis framework (LIANA) was used to infer ligand–receptor interactions^47^. Following the selection of AE, CM, HPC, EPP, MES and PE/ST clusters, the top 30 ligand (source)–receptor (target) interactions were visualized (Extended Data Fig. 8a,b). The most frequent interactions (aggregate_rank ≤ 0.01) were observed in BG-HFOs between CM and AE, CM and PE/ST, PE/ST and AE, and MES and AE (Extended Data Fig. 8a).

Designating the cluster source (ligand provider; magnitude rank < 0.01) and the cluster target (receptor-expressing; magnitude rank < 0.01), we performed gene-set enrichment analysis using Enrichr and the Gene Ontology Biological Process 2023 database^48,49^.

The CM cluster showed expression of ligands that in the AE and PE/ST clusters positively regulate processes of extracellular matrix and structure organization, collagen fibril organization, cell adhesion, proliferation, migration and integrin-mediated signalling. By ligands expressed from both the PE/ST and MES clusters, similar processes were positively regulated in the AE. Notably, the MES cluster seems to positively regulate processes of angiogenesis sprouting and blood vessel/EC migration in the AE. Accordingly, in response to the signalling from MES, AE upregulated expression of receptors involved in the same processes, that is, cell motility, migration, angiogenesis and blood vessel sprouting (Extended Data Fig. 8c).

Furthermore, in response to signalling from CM and PE/ST, the AE showed expression of receptors regulating cell migration and motility. However, only the CM cluster is the origin of positive regulation of calcium-mediated signalling, vasculogenesis, vascular smooth muscle cell, cardiac atrium, and ventricular trabecular myocardium development and morphogenesis in the AE. Notably, the PE/ST cluster, in response to signalling from CM, upregulated expression of receptors involved in aorta development and pulmonary valve morphogenesis (Extended Data Fig. 8c).

All haematopoietic waves originate from specialized HE, which derives from and maintains aspects and gene expression patterns of the AE^37–39^. Considering that the AE cluster in BG-HFOs possibly includes scattered HE of different haematopoietic waves, ligand–receptor interactions between the AE and the haematopoietic progenitor clusters (putatively representing definitive haematopoiesis/HPC and primitive haematopoiesis/EPP) were investigated. Gene-set enrichment analysis suggests that AE directed in both (EPP and HPC) cellular processes of extracellular matrix organization, cellular proliferation, extravasation and protein modification processes such as phospho/dephosphorylation. Moreover, the analysis of AE-to-EPP signalling indicates a role of AE in regulating processes of EC migration and angiogenesis sprouting (Extended Data Fig. 8d). The corresponding receptors expressed by both the HPC and EPP clusters (in response to the AE signalling) included genes regulating cell motility, migration, positive regulation of protein phosphorylation, regulation of cell-matrix adhesion and the integrin-mediated signalling pathway; the latter was the most upregulated process in the HPC cluster in response to AE (Extended Data Fig. 8d).

Together, the CCC analysis enabled the identification of cell/tissue communication in BG-HFOs, including well-known signalling in native embryogenesis of the cardiovascular and haematopoietic system.

### BG-HFOs display erythro-myeloid and lymphoid potency

**Fig. 5:**
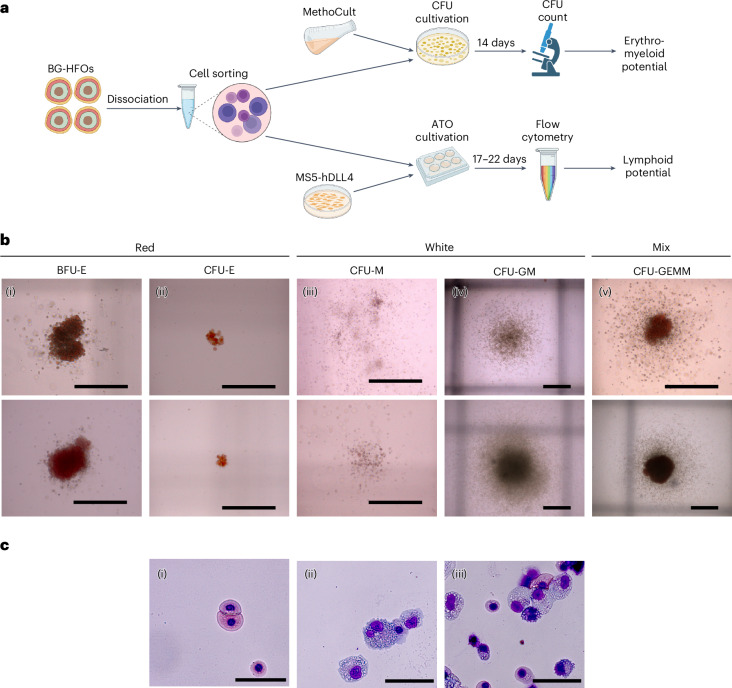
BG-HFO-derived HPCs display erythro-myeloid potential. **a**, Experimental design. First, 40–80 BG-HFOs were dissociated and pooled. CD34^+^ cells were sorted to assess erythro-myeloid (CFU assay; top) and lymphoid (ATO assay; bottom) potential. Purified populations (CD34^+^CD43^+^, CD34^+^CD43^−^ and CD34^−^CD43^+^) were sorted to further assess the erythro-myeloid potential. **b**, Erythro-myeloid potential. Representative images of (i) burst-forming unit-erythroid (BFU-E), (ii) CFU-erythroid (CFU-E), (iii) CFU-macrophage (CFU-M), (iv) CFU-granulocyte–macrophage (CFU-GM) and (v) CFU-granulocyte–erythroid–macrophage–megakaryocyte (CFU-GEMM) colonies derived from CD34^+^ cells after 14 days of culture in MethoCult. **c**, Erythro-myeloid cell morphology. May–Grünwald–Giemsa staining of CFU assay-derived (i) red colonies composed of erythroid cells, (ii) white colonies composed of myeloid cells and (iii) mix colonies composed of erythroid and myeloid cells. Scale bars, 500 µm (**b**) and 50 µm (**c**).

Magnetic-activated cell sorted CD34^+^ haemato-endothelial cells from d10–d12 BG-HFOs were tested for their erythroid and myeloid potential using colony-forming-unit (CFU) assays (Fig. 5a). Formation of ‘red colonies’, including dark red burst-forming-unit-erythroid cells (Fig. 5b(i)) and smaller light-red colonies identified as CFU-erythroid (Fig. 5b(ii)), suggested the presence of erythroid cells of different maturity.

‘White colonies’ of the myeloid lineage included clusters of grey round cells categorized as CFU-macrophages (Fig. 5b(iii)) and colonies with a dense and dark grey core supposedly composed of macrophages and small bright granulocytes (CFU-macrophages–granulocytes; Fig. 5b(iv)). In addition, ‘mixed colonies’ of granulocytes, erythroblasts and macrophages (Fig. 5b(v)) were detected.

Colony composition was confirmed by May–Grünwald–Giemsa staining: the red colonies were round and had reddish cytoplasm and compact nuclei underlining their erythroid phenotype (Fig. 5c(i)), whereas the white colonies were composed of big irregular-shaped cells with lobulated nuclei typical of macrophages and monocytes (Fig. 5c(ii)). In mixed colonies, the presence of erythroid cells, macrophages and smaller cells with cytoplasmic protrusions typical of activated macrophages and dendritic cells (Fig. 5c(iii)) was observed.

**Fig. 6:**
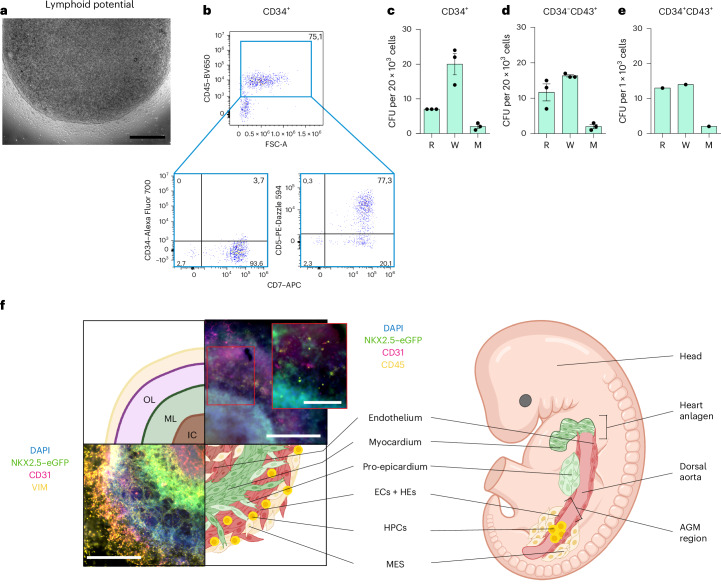
BG-HFO-derived HPCs display lymphoid potential. **a**, Representative image of a d22 ATO surrounded by haematopoietic cells, including T cell progenitors. **b**, Representative flow cytometry plots of cells derived from 2–6 ATOs generated using CD34^+^ cells at d17 of ATO differentiation showing the generation of CD34^−^CD45^+^CD5^+^CD7^+^ T cell progenitors (*n* = 2). The percentage of cells in the different quadrants is indicated. **c**–**e**, Number of red (R), white (W) and mix (M) colonies per 20,000 BG-HFO-derived CD34^+^ cells (*n* = 3; **c**), 20,000 BG-HFO-derived CD34^−^CD43^+^ HPCs (*n* = 3; **d**) and 1,000 BG-HFO-derived CD34^+^CD43^+^ HPCs (*n* = 1; **e**) seeded in MethoCult. Data are presented as the mean ± s.e.m. **f**, Schematic of the front view of a typical BG-HFO shown compared with the cross-section of a human embryo showing heart and haematopoietic anlagen. Whole-mount immunofluorescence images of two hES3 NKX2.5–eGFP-derived BG-HFOs stained with DAPI, anti-CD31 and anti-VIM (bottom left) or anti-CD45 (top right) were incorporated. Inset: Higher magnification image of the region in the red box to allow better visualization of the position of CD45^+^ cells. Scale bars, 500 µm (**a**,**f** (main images)) and 200 µm (**f**(inset)).

**Extended Data Fig. 9:**
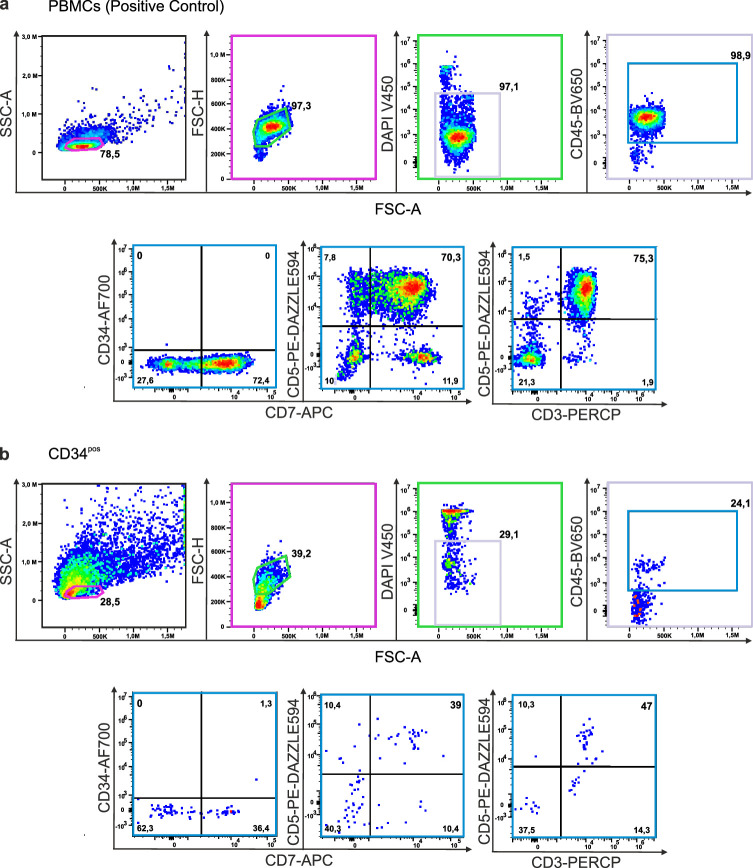
Flow cytometry allows for the identification of ATO-derived T cells. **a**,**b**. Representative gating strategy and flow cytometry plots of peripheral blood mononucleated cells (**a**, PBMCs, positive control) and cells derived from 2–6 ATOs generated using CD34^pos^ cells at day 44 of ATO differentiation (**b**) showing generation of CD34^neg^/CD45^pos^/CD5^pos^/CD7^pos^/CD3^pos^ T cells (n = 1).

To investigate the lymphoid potential, artificial thymic organoid (ATO) assays were performed^50,51^: BG-HFO-derived CD34^+^ haemato-endothelial cells were aggregated with MS5-hDLL4 cells (providing the NOTCH signalling for lymphoid differentiation) and subjected to an air–liquid interface culture for 17–22 days (Fig. 5a). Analysis of released cells (Fig. 6a) showed a fraction of CD45^+^CD34^−^ phenotype expressing the T cell progenitor markers CD5 and CD7 (Fig. 6b). Maintenance of ATOs for up to 44 days allowed for further maturation of a minor portion of T cell progenitors to CD3-expressing T cells; the gating strategy was based on peripheral blood mononucleated cells (Extended Data Fig. 9a,b).

CD43 is one of the earliest markers expressed in CD34^+^ committed haematopoietic cells. Therefore, CD34^−^CD43^+^, CD34^+^CD43^+^ and CD34^+^CD43^−^ cells were enriched for erythro-myeloid potential assessment through fluorescence-activated cell sorting. Quantification of CD34^+^-derived colonies revealed greater numbers of white colonies compared with red colonies and only a few of mixed composition (Fig. 6c); CD34^−^CD43^+^ HPCs showed a similar potential of colony formation (Fig. 6d). Notably, the use of CD34^+^CD43^+^ HPCs resulted in a 20-fold higher efficiency in the CFU assay given that similar numbers of colonies were obtained from fewer (20-fold less) seeded cells—that is, 20,000 CD34^+^ haemato-endothelial cells (Fig. 6c) or CD34^−^CD43^+^ HPCs (Fig. 6d) versus 1,000 CD34^+^CD43^+^ HPCs (Fig. 6e). As expected, the sorted CD34^+^ but CD43-depleted cell population did not form any colonies in the CFU assay, probably representing the CD34^+^ endothelial component in BG-HFOs. These data suggest that the haematopoietic multipotential is retained by the CD43-expressing HPCs; CD43^+^ cells co-expressing CD34, which represent an early fraction of HPCs, retain the majority of this potential.

Together, BG-HFO-derived haemato-endothelial cells displayed erythroid, myeloid and lymphoid multipotency; the subset of CD34^+^CD43^+^ HPCs displayed the highest erythro-myeloid potency.

## Discussion

Due to its complexity and inaccessibility, knowledge on native human haematopoietic development, its regulating mechanisms and functional interplay with neighbouring tissues during embryogenesis remains poorly understood, calling for advanced in vitro models.

Based on the stage-specific modulations of HFO differentiation by factors known to stimulate HE formation and haematopoiesis from hPSCs^19–21,36^, we here presented a highly structured multi-tissue organoid model termed BG-HFO, which recapitulates key aspects of the simultaneous cardiac and haemato-vascular development in the embryo^52^.

Although BG-HFOs recapitulate the layered multi-tissue structure of HFOs—particularly the myocardial layer—differences include a major loss of endoderm anlagen and the endocardial-like layer but the gain of VE, AE and HE; the latter localized in the outer layer of BG-HFOs where haematopoietic cells were also predominantly detected (Fig. 2g–i and Extended Data Figs. 4d,e, 6).

A schematic of similarities between the BG-HFO model and cardiac and haematopoietic development in the embryo is shown in Fig. 6f. In human development both the splanchnic mesoderm-derived heart fields and the primary aortic tubes fuse into a single heart tube and one dorsal aorta at around three weeks of gestation; WNT pathway activation and factors such as VEGF, bFGF and BMP4, provided by the foregut endoderm and lateral plate mesoderm, drive dorsal aorta formation and haematopoiesis^11,53–55^.

Simultaneously, ventrally located mesenchyme will specialize into PE/ST supporting heart development^52,55^. In BG-HFOs about 20% of cells were specified as PE/ST mesenchyme; thus, signals directing vascular and haematopoietic differentiation and spatial localization are probably provided by both the WNT-induced self-organizing endogenous tissues (that is, the ML and MES) and exogenous components from the stage-specific BOOST.1–4 treatments. Distinct endothelial subtypes (that is, VE and HE/AE) were observed in BG-HFOs (Figs. 2a, 3c and 4a); however, scRNA-seq analysis showed no clear separation of AE from HE, apparently due to the high degree of expression similarities. Nevertheless, immunofluorescence staining allowed for the identification of ALDH1A1^+^CD31^+^ and RUNX1^+^CD31^+^ HE in the outer layer, confirming the presence of a HE population located in this morphologically distinct area (Extended Data Fig. 6).

A cluster of HPCs characterized by co-expression of *CD44*, *SPN*, *PTPRC*, *RUNX1*, *MYB*, *GATA2* and *TAL1* (refs. ^36,42^) was detected. Moreover, CD45^+^CD31^+^ and CD43^+^CD31^+^ HPCs (and more mature derivatives of the CD45^+^, CD43^+^, CD14^+^ or CD4^+^ phenotype) were also observed prevalently in the outer layer (Fig. 2g–i and Extended Data Fig. 4c–f). Intermingling of haematopoietic cells with the endothelium in the outer layer may represent a functional equivalent of the native HE in the embryo giving rise to haematopoiesis. In our in vitro model the mesenchyme surrounding the endothelial/haematopoietic layer in the outer layer (Fig. 1j and Extended Data Fig. 3a,b) may provide instructive signals equivalent to the subaortic mesenchyme outside and within the AGM region in vivo^56^, thus promoting and specifying the spatially defined endothelial and haematopoietic development in BG-HFOs (Fig. 6f).

A cluster of early primitive progenitors (possibly progenitors of primitive haematopoiesis), megakaryocytes, erythroid derivatives, macrophages and monocytes were also detected.

Calvanese and colleagues recently mapped the transcriptome of HE and HSC development and maintenance in humans^42^. Notably, aspects of these expression patterns are reflected by the BG-HFO-derived HPC cluster. This includes, by degrees, expression of the proliferation-related genes *TOP2A* and *MKI67* as well as *GFI1*, *ITGA2B*, *SMIM24*, *STAT5A* and *KCNK17* (enriched in the overall HSC population in vivo), expression of *MLLT3* and *MECOM* (known to be involved in HSC self-renewal), and the potential human AGM-HSC markers *RAB27B* and *ALDH1A1*, which are expressed in HE and emerging HSCs in the AGM region of the embryo (Fig. 4a).

On the other hand, important key markers recently discovered in HSCs (such as *HOXA9*, *SPINK2* and *HLF*) and HE (such as *IL33*) were not detectable in the HPC and AE of our in vitro model, indicating a lack of some aspects of bona fide HSC-competent HE and HSCs, at least at the BG-HFO stage analysed in this study^42^.

The presence of effective multipotent haematopoietic progenitors in our model was underlined by functional readouts: CFU assays revealed the formation of erythroid and myeloid colonies, where the majority of the erythro-myeloid potential was retained by the CD34^+^CD43^+^ HPC population.

Using ATO assays^57^, we demonstrated lymphoid potential by generation of CD34^−^CD45^+^CD5^+^CD7^+^ T cell progenitors by day 17 of differentiation (Fig. 6b) and a few CD3-expressing T cells by d44 of differentiation (Extended Data Fig. 9b); expression of *BCL11A* and *CD7* by some of the HPCs further emphasises the presence of cells with lymphoid potential (Extended Data Fig. 7g).

During embryonic development, as the three haematopoietic waves occur, the expression of the embryonic *HBZ* and *HBE1*, fetal *HBA2* and *HBG1*, and adult *HBA2* and *HBB* globin genes also switches^58,59^. Globin gene expression in erythroid derivatives of BG-HFOs showed the embryonic/fetal gene signature and, as expected, very low expression of the adult pattern (Extended Data Fig. 7d), which is activated only at later stages of fetus development. Thus, the BG-HFO signature seems consistent with the first weeks of native human haematopoiesis, where primitive and definitive waves overlap^59^.

In summary, the expression of embryonic globin genes and the high erythro-myeloid potential reflect the presence of primitive haematopoiesis in our model^9,10^; however, a considerable part of BG-HFO-derived erythroid cells express fetal globin genes, a feature of definitive (HSC-independent and -dependent) haematopoiesis. These findings hint towards the simultaneous presence of primitive and definitive haematopoiesis in our model, considering that primitive haematopoiesis is limited to the erythro-myeloid potential^9^, whereas lymphoid potential was also proven in BG-HFOs.

Definitive haematopoiesis was further corroborated by annotations to the ‘BlueprintEncodeData’ database, which indicates the presence of different types of progenitors, including common myeloid progenitors, common lymphoid progenitors, granulocyte–macrophage progenitors and megakaryocyte–erythroid progenitors, in BG-HFOs (Extended Data Fig. 7f). However, whether this represents transient or also HSC-dependent haematopoiesis needs future investigation^7,37–39^.

The most remarkable observations from CCC analysis showed the role of MES in supporting AE development, enhancing processes of cell migration, angiogenesis and blood vessel sprouting (Extended Data Fig. 8c), potentially corroborating the role of the subaortic mesenchyme in endothelial-blood development in vivo^56^. Moreover, CCC analysis suggested a contribution of the AE to heart development (Extended Data Fig. 8c), consistent with these tissues being anatomically and developmentally correlated in vivo^53,55^. Notably, in response to CM cluster signalling, positive regulation of aorta development and pulmonary valve morphogenesis was observed in the PE/ST cluster (Extended Data Fig. 8c), suggesting a putative role of PE/ST mesenchyme in the development of these neighbouring tissues.

The AE cluster also seems to regulate extracellular matrix organization, cell proliferation and migration in both the HPC and EPP clusters. However, AE-mediated regulation of processes such as EC migration and angiogenic sprouting was only observed in the EPP cluster, representing progenitors of the primitive haematopoiesis known to retain angiogenic potential^4,37,39^ (Extended Data Fig. 8d).

Different models recapitulating various aspects of haematopoietic development and related tissues have been recently published. Applying mouse ESCs, Rossi and colleagues published a gastruloid model recapitulating gastrulation and cardiogenesis^60^ and later showed the presence of haematopoietic derivatives putatively from primitive haematopoiesis given that lymphoid potential was not demonstrated^26^.

Chao and colleagues described cell–cell interactions and pathways involved between embryonic tissues from the three germ layers, extra-embryonic tissues and primitive haematopoietic derivatives in human embryonic organoids^27^. The human embryonic organoid system models early embryonic development at the stage of germ-layer specification, whereas aspects of more mature tissue formation such as cardiac or definitive haematopoiesis are lacking. Models recapitulating aspects of the bone-marrow niche, rather resembling the adult site of definitive haematopoiesis, have also been generated^61,62^. In comparison, we here provide a human in vitro model simultaneously recapitulating the co-development of heart and vascular anlagen together with key aspects of complex multiwave and multipotent haematopoiesis, recapitulating aspects of spatial self-organization known from native human development^3,6,10,11^.

**Extended Data Fig. 10:**
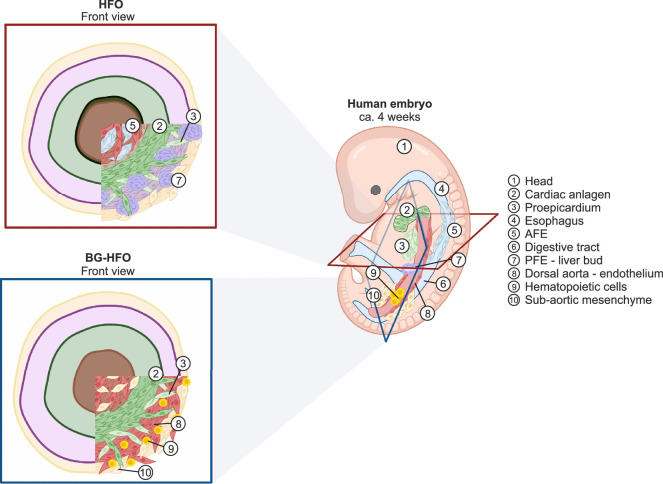
Scheme illustrating the analogy of HFOs and BG-HFOs compared to the native human embryo. Hypothesized comparison of the front view of an HFO and a BG-HFO with the sagittal plane of an early human embryo (ca. week 4 of development). While HFOs recapitulate aspects of the embryonic heart and foregut region, a relatively anterior area of the embryo, BG-HFOs recapitulate aspects of the heart, dorsal aorta-like tissue, and hematopoiesis, resembling an area of the embryo that is rather postero-ventral.

Notably, our previous HFO model resembles an anterior region of the embryo (that is, heart and foregut). In comparison, BG-HFOs show an expanded PE/ST compartment in addition to the myocardial tissue and present elements resembling an AGM-like region but absence of foregut endoderm. Thus, our latter model represents a more postero-ventral area of embryo development as summarized in Extended Data Fig. 10. The axis shift is probably the result of the addition of growth factors, specifically BMP4 and bFGF, at early time points. The supplementation of BMP4 at d−2 and d0 activates the WNT pathway in a cascade^63,64^, resulting in mesodermal pre-priming, which is reinforced by CHIR supplementation on d0 and leads to endodermal loss. An organoid model recapitulating proepicardium and epicardium development was recently described (‘epicardioids’)^65^. The protocol is based on mid-anterior primitive streak, cardiac mesoderm and vascular induction achieved by early WNT pathway activation combined with BMP4 and bFGF at early time points. This work seems to corroborate our hypothesis that the combined pathway activation at early stages plays an instructive role in the pro-epicardial tissue expansion and postero-ventral phenotype in BG-HFOs (Extended Data Fig. 10).

Together, the generation of BG-HFOs demonstrates the plasticity of the HFO model and a valid strategy for recapitulating different, yet morphologically and physiologically well-defined embryonic anlagen by specific protocol modifications. BG-HFOs represent a potent tool to study the cellular and molecular properties as well as mechanisms of early haematopoiesis and cardiogenesis, which are inaccessible in the human embryo. Due to its multi-tissue composition, robustness and reproducibility, the model also provides an advanced screening platform for modelling diseases and investigating drugs, teratogens and toxins.

## Methods

Our research complies with all relevant ethical regulations. Experiments using hESCs lines were performed under allowance ‘108 Genehmigung nach dem Stammzellgesetz’ granted by the Robert Koch Institute.

### Cell culture, HFO and BG-HFO formation

Both hES3 NKX2.5–eGFP^66,67^ and HSC_ADCF_SeV-iPS2 (ref. ^33^) cells were cultured on irradiated embryonic mouse fibroblasts in an incubator at 37 °C and 5% CO_2_. At 80% colony confluence, the cells were passaged and either seeded onto fresh irradiated fibroblasts or, to start organoid differentiation, transferred to Geltrex-coated flasks in Essential 8 (E8) medium (DMEM/F12, HEPES with 64 mg I^−1^ ascorbic acid 2-phosphate, 100 μg l^−1^ bFGF, 20 mg l^−1^ insulin, 543 mg l^−1^ NaHCO_3_, 14 μg l^−1^ Na_2_SeO_3_, 10.7 mg l^−1^ transferrin and 2 μg l^−1^ TGFβ) supplemented with 10 μM Y-27632 (Tocris). The cells were passaged every 3–4 days and the medium was changed daily except the day immediately after the passaging. A previously published protocol^29^ was used to generate HFOs. To generate BG-HFOs, the HFO protocol was adapted by the addition of cytokines at specific time points; the detailed step-by-step protocol can be found at 10.17504/protocols.io.e6nvw1nkdlmk/v1. Briefly, were detached from Geltrex-coated flasks on d−4 cells using Accutase (Thermo Fisher Scientific) and seeded (5 × 10^3^ cells per well) in E8 medium supplemented with 10 μM Y-27632 in a U-shaped ultralow-attachment 96-well plate (Thermo Fisher Scientific and FaCellitate). The plate was centrifuged at 300*g* and 4 °C for 6 min and then placed in the incubator to let one aggregate per well form overnight. Each aggregate was embedded in a Matrigel droplet (Corning, catalogue number 354234) on d−2. Matrigel lot numbers 100007, 1335001, 1336002, 1013001, 1335001, 1336002, 2033002 and 2237001 were applied. After embedding, the plate was placed in an incubator at 37 °C for 50 min to let the Matrigel solidify. E8 medium supplemented with 10 ng ml^−1^ BMP4 (Peprotech) was then added on top of the Matrigel-embedded aggregates. On d0, the E8 medium was replaced with RPMI 1640 medium (Thermo Fisher Scientific) containing 2% B-27 supplement without insulin (RB−; Thermo Fisher Scientific) and supplemented with 7.5 μM CHIR (synthesised by the Institute for Organic Chemistry, Leibniz University Hannover), 10 ng ml^−1^ BMP4 and bFGF 5 ng ml^−1^ (Peprotech). After 24 h, on d1, the medium was exchanged with RB− supplemented with 50 ng ml^−1^ VEGF (Peprotech) and 10 ng ml^−1^ bFGF. On d3, RB− supplemented with 5 μM IWP2 (Tocris), 50 ng ml^−1^ VEGF, 10 ng ml^−1^ bFGF, 100 ng ml^−1^ SCF (Peprotech), 17 ng ml^−1^ EPO (Peprotech), 10 ng ml^−1^ IL-6 (Peprotech), 10 ng ml^−1^ IL-11 (Peprotech) and 25 ng ml^−1^ IGF-1 (Peprotech) was added. Following incubation for 48 h, on d5, the medium was exchanged with RB− medium supplemented with the same molecules in the d3 medium plus 30 ng ml^−1^ TPO (Peprotech), 10 ng ml^−1^ FLT3 (Peprotech), 30 ng ml^−1^ IL-3 (Peprotech), 10 ng ml^−1^ BMP4 and 20 ng ml^−1^ SHH (Peprotech). From d7 onwards, the BG-HFOs were cultivated in RPMI 1640 medium containing 2% B-27 supplement (RB +; Thermo Fisher Scientific) supplemented with the same cytokines added on d5. The cultivation medium was further supplemented with 1:100 penicillin–streptomycin (Sigma Aldrich). Differentiation was completed on d10; BG-HFOs were kept in culture for downstream analysis until d14. Images of whole HFOs and BG-HFOs were taken using an Axio Observer A1 (Zeiss) or Olympus CKX41 inverted microscope (Olympus); images were processed using the AxioVision Se64 Rel. 4.8 software.

### Whole-mount immunofluorescence staining

BG-HFOs were washed in 1×PBS without Mg^2+^ and Ca^2+^ (PBS w/o), fixed with 4% paraformaldehyde overnight at 4 °C, washed with Tris-buffered saline (TBS) and incubated overnight with blocking buffer (TBS containing 5% BSA and 0.25% Triton X-100) at 4 °C. Incubation with primary antibodies was performed in staining buffer (TBS containing 1% BSA) for four days at 4 °C; the antibody solution was renewed after two days. The following primary antibodies were used at the indicated dilutions: anti-CD31 (Agilent, JC70A; 1:20), anti-CD31 (Abcam, ab28364; 1:200), anti-cTnT (goat, Abcam, ab64623; 1:100), anti-cTnT (rabbit, Abcam, ab209813; 1:300), anti-cTnT (mouse, Thermo Fisher Scientific, MA5-12960; 1:100), anti-CD45 (Abcam, ab8216; 1:100), anti-NKX2.5 (Cell Signaling Technology, 8792; 1:800), anti-vimentin (Abcam, ab92547; 1:400), anti-ALHD1A1 (Santa Cruz Biotechnology, sc-374149; 1:1,000), anti-CD43 (Abcam, ab101533; 1:200) and anti-CD14 (Abcam, ab182032; 1:200). The BG-HFOs were then washed twice (1 h each wash) with TBS at 4 °C and incubated with secondary antibodies in staining buffer containing 0.57 μg ml^−1^ DAPI, to label the cells nuclei, for two days at 4 °C. The following secondary antibodies were used at the indicated dilutions: donkey anti-rabbit Alexa Fluor 647 (Jackson ImmunoResearch, 711-606-152; 1:500), donkey anti-rabbit Alexa Fluor 488 (Jackson ImmunoResearch, 711-545-152; 1:500), donkey anti-goat Alexa Fluor 488 (Jackson ImmunoResearch, 705-545-147; 1:500), donkey anti-mouse Cy3 (Jackson ImmunoResearch, 715-165-150; 1:200) and donkey anti-goat Alexa Fluor 647 (Jackson ImmunoResearch, 705-605-147; 1:200) conjugates. The BG-HFOs were washed three times (1 h each wash) with TBS at 4 °C. Images were taken on an Axio Observer A1 microscope (Zeiss). Images were processed utilizing the following programs: AxioVision Se64 Rel. 4.8 and/or ImageJ 1.52p; potential adjustments were applied to the entire image. For confocal microscopy, tissue clearing of BG-HFOs was performed. BG-HFOs were dehydrated though incubation (1 h for each dilution) in increasing stepwise dilutions of ethanol in water (25, 50 and 75%) at room temperature. Finally, the BG-HFOs were dehydrated with 99% ethanol overnight at room temperature. The following day the organoids were incubated in increasing concentrations (25, 50 and 75%) of a 1:1 solution of methyl salicylate and benzyl benzoate in ethanol (1 h per dilution), and finally overnight in 100% of the 1:1 methyl salicylate:benzyl benzoate at 4 °C. Imaging of cleared BG-HFOs was performed using a Zeiss 980 Airyscan confocal microscope, and the Zen 3.5 software for the acquisition and Zen 2.6 for the analysis.

### Cryosectioning and staining

The culture medium of BG-HFOs was supplemented with 0.5 mg ml^−1^ dextran (Sigma Aldrich) one day before cryoembedding. The BG-HFOs in U-bottomed 96-well plates were then washed with PBS w/o and overlaid with Tissue-Tek (Sakura Finetek). Using a small spatula, the organoids were transferred to a cryomold filled with Tissue-Tek, visible bubbles were removed with a 200 µl pipette and the sample was frozen in a Microm HM 560 cryotome (Thermo Fisher Scientific). The BG-HFOs were stored frozen at −80 °C until use. The organoids were sectioned (10 μm sections) using a Microm HM 560 cryotome, the sections were collected on glass slides, dried overnight at room temperature and stored at −80 °C until use. For immunofluorescence staining, the cryosections were fixed with 4% paraformaldehyde for 5 min at room temperature, followed by incubation with blocking buffer for 1 h at room temperature and incubation with primary antibodies diluted in staining buffer overnight at 4 °C. The following primary antibodies were used at the indicated dilutions: anti-CD31 (Agilent, JC70A; 1:20), anti-CD144 (Abcam, ab33168; 1:500), anti-cTnT (goat, Abcam, ab64623; 1:100), anti-cTnT (rabbit, Abcam, ab209813; 1:300), anti-CD43 (Abcam, ab101533; 1:200), anti-CD45 (Abcam, ab8216; 1:100) and anti-RUNX1 (Abcam, ab240639; 1:1,000). The following day, the cryosections were washed in 1×TBS and incubated with secondary antibodies diluted in staining buffer for 1 h at room temperature. The following secondary antibodies were used at the indicated dilutions: donkey anti-rabbit Alexa Fluor 488 (Jackson ImmunoResearch, 711-545-152; 1:500), donkey anti-goat Alexa Fluor 488 (Jackson ImmunoResearch, 705-545-147; 1:500) and donkey anti-mouse Cy3 (Jackson ImmunoResearch, 715-165-150; 1:200) conjugates. Next, the cryosections were stained with 1.7 μg ml^−1^ DAPI for 15 min at room temperature to label the nuclei, mounted with mounting medium (Dako), covered with a coverslip and kept at room temperature overnight before analysis. The stained sections were stored at 4 °C. Images were taken on an Axio Observer A1 (Zeiss) and processed using the AxioVision SE64 Rel. 4.8 and ImageJ 1.52p software.

### Flow cytometry on HFOs and BG-HFOs

HFOs and BG-HFOs were individually washed in PBS w/o, transferred to V-bottomed 96-well plates (Boettger), dissociated into single cells using a STEMdiff cardiomyocyte dissociation kit (Stemcell Technologies), applying 100 µl Cardiomyocyte Dissociation Medium for 12 min at 37 °C. Four or five dissociated organoids were pooled and redistributed again to four or five wells for analysis homogeneity, washed with 100 µl Flow Buffer (PBS w/o supplemented with 0.5% BSA), and fixed and stained using a Fix&Perm kit (Nordic MUbio). The following antibodies were used (all at 1:50; Miltenyi Biotec; catalogue numbers are indicated in parentheses): anti-human CD31–allophycocyanin (APC) (130-092-652), anti-human CD31–phycoerythrin (PE) (130-119-142), anti-human CTNT–APC (130-106-689), anti-human CD90–VioBlue (130-114-866), anti-human CD34–VioBlue (130-124-459), anti-human CD43–APC-Vio770 (130-114-596), anti-human CD45–PE-Vio770 (130-110-634), anti-human CD144–APC (130-125-985), anti-human CD73–APC (130-112-061) and anti-human CD4–PE-Vio670 (130-132-907) conjugates. Numerical source data used for the flow cytometry-based statistical analyses are reported in Supplementary Table 1(i),(iii),(v). The gating strategy was built on isotype controls and unstained controls. The following isotype controls (all at 1:50; Miltenyi Biotec; catalogue numbers are indicated in parentheses) were used: anti-human IgG1–PE (130-118-347), anti-human IgG1–VioBlue (130-113-442), anti-human IgG1–APC-Vio770 (130-113-435), anti-human IgG1–PE-Vio770 (130-113-440) and anti-human IgG1–APC (130-113-434). Cells were measured on a MACSQuant Analyzer 10 (Miltenyi Biotec) or CytoFLEX S (Beckmann Coulter) system; data were acquired using the MACSQuant Analyzer 10 software and analysed using the FlowJo v10 software.

### Cell sorting

BG-HFOs (40–80 organoids per sort) were individually washed in PBS w/o, transferred to a new V-bottomed 96-well plate (Thermo Fisher Scientific) and dissociated into single cells using the STEMdiff cardiomyocyte dissociation kit, with 100 µl Cardiomyocyte Dissociation Medium (Stemcell Technologies) for 12 min at 37 °C. Next, the BG-HFOs were pooled and washed with 3× the volume of RB+ medium. To sort cells positive for the marker CD34, single cells were labelled with a CD34 Microbead Kit UltraPure (Miltenyi Biotec, 130-100-453) and magnetically sorted through M columns (Miltenyi Biotec) with an OctoMACS separator (Miltenyi Biotec). The BG-HFO-derived CD34^+^ cells were washed and resuspended in RB+ medium, strained using a 70 µm cell strainer, counted, analysed by flow cytometry and immediately used for downstream analysis. To sort CD34^+^CD43^+^, CD34^−^CD43^+^ and CD34^+^CD43^−^ cells, single cells were labelled with anti-CD34-VioBlue (Miltenyi Biotec, 130-124-459; 1:50) and anti-CD43-APC-Vio770 (Miltenyi Biotec, 130-114-596; 1:50) for 10 min at 4 °C, washed with RB+ medium and FACS sorted using a BD FACSAria III Fusion cell sorter. After sorting, the cells were collected in RB+ medium.

### CFU assay and May–Grünwald–Giemsa staining

To test the erythro-myeloid potential of HPCs, BG-HFO-derived CD34^+^ (20 × 10^3^ or 40 × 10^3^), CD34^+^CD43^+^ (1 × 10^3^ or 4 × 10^3^), CD34^−^CD43^+^ (20 × 10^3^) or CD34^+^CD43^−^ cells (20 × 10^3^) were sorted from 40–80 dissociated and pooled BG-HFOs, as described above. RB+ medium was added to the cells to a total volume of 100 µl and the samples were vortexed for 5 s into 1.1 ml MethoCult (Stemcell Technologies) before seeding in SmartDish six-well culture plates (Stemcell Technologies). To allow constant humidification, 4 ml sterile water was placed in each gap of the six-well plate, and the plate was placed into a 245 mm Square Dish, Non-treated (Corning) dish containing four small 3.5 ×10 mm culture dishes filled with sterile water. The plates were incubated at 37 °C for 14 days, and the colonies were counted and morphologically characterized using an Axio Observer A7 (Zeiss). Colony counts are reported in Supplementary Table 1(ii). If needed, the plates were maintained in culture for up to one week in humidified conditions at 30 °C. When needed, the colonies were manually picked up using a 200 µl pipette and washed with PBS w/o. The cells then were collected on glass slides via Cytospin 2 (Shandon) centrifugation (55*g* for 9 min), dried overnight and stored at room temperature. The cells were stained for 5 min at room temperature with 1:1 May–Grünwald stain (Sigma Aldrich), washed in deionized water and then stained with 1:20 Giemsa staining for 20 min. After staining, the cells were washed with deionized water until the water was clean and dried at room temperature. Thereafter, the glass slides were mounted with Roti-histokitt (ROTH), covered with a coverslip and kept at room temperature overnight before analysis. A total of *n* = 3 biologically independent experiments were performed for each group of analysed cells (BG-HFO-derived CD34^+^, CD34^−^CD43^+^ and CD34^+^ CD43^−^ cells), excepting CD34^+^CD43^+^ cells, where *n* = 1. Morphological characterization and imaging of colonies and cells were performed using an Axio Observer A7 microscope (Zeiss) and processed using the AxioVision SE64 Rel. 4.8 or Zen 3.5 software.

### T cell progenitor differentiation and assessment via ATO assay and flow cytometry

MS5 murine stromal cells^68^ were transduced with the third-generation lentiviral vector encoding human Delta-like 4 (DLL4), wherein the hDLL4 was cloned in the pRRL.PPT.SFFV vector backbone combined with IRES-puromycin expression in the same construct. The transduced cells were selected with 5 µg ml^−1^ puromycin and characterized using anti-hDLL4-PE (BioLegend, 346505).

The MS5-hDLL4 cells were cultured in reconstituted MEM α medium (Thermo Fisher Scientific, 12000-014) supplemented with 10% FBS Brazil one (PAN-Biotech, P30-3309), 2 mM sodium pyruvate (PAN-Biotech, P04-43100), 2 mM l-glutamine (PAN-Biotech, P04-80050) and 1:100 penicillin–streptomycin (PAN-Biotech, P06-07100).

To initiate ATOs, 1 × 10^4^ CD34^+^ cells derived from the BG-HFOs were mixed with 0.15 × 10^6^ MS5-hDLL4 cells for each ATO. The resulting cell slurry was resuspended in RPMI 1640 medium (PAN-Biotech, P04-16500) supplemented with 4% B-27 supplement (Gibco, 17504-044) and 30 µM ascorbic acid (Sigma Aldrich, A8960-5G) plus 2.5 ng ml^−1^ human interleukin-7 (Peprotech, 200-07-100UG), 5 ng ml^−1^ human FMS-related tyrosine kinase ligand (Peprotech, 300-19-100UG) and 5 ng ml^−1^ human SCF (Peprotech, 300-07-100UG). The cells were centrifuged for 5 min at 4 °C in a swinging-bucket rotor, the supernatant was almost completely removed and the formed aggregate of cells was resuspended in a total volume of 5 µl of the remaining medium per number of desired ATOs. Using a P20 pipette, 5 µl of the cell mixture, corresponding to one ATO, was transferred to 0.4 µm cell culture inserts (Merck Millipore, PICMORG50), which was placed in six-well plates with 1 ml of the complete RPMI medium in each well^50^.

The plate was incubated and the medium was exchanged every 4–5 days. After 17–22 days, and up to 44 days, cells surrounding the ATOs were collected and the ATOs were mechanically pipetted and dissociated. The membrane inserts were washed with MACS buffer (0.5% BSA (PAN-Biotech, P06-139350) + 2 mM EDTA (Thermo Fisher Scientific, 15575-038) in PBS), the cells were strained through a 70 µm cell strainer to filter and exclude the stromal cells, and further washed and centrifuged at 300*g* for 5 min at 4 °C. The cells were resuspended in MACS buffer containing antibodies and FcR Block (Miltenyi Biotec, 130-059-901), and incubated at 4 °C for 20 min in the dark. Markers to detect presence of T cell progenitors were used^51^. The following antibodies were applied: anti-human CD33–brilliant violet (BV)421 (clone P67.6; BioLegend, 366622), anti-human CD14–BV421, (clone 63D3; BioLegend, 367144), anti-human CD235ab–Pacific blue (clone HIR2; BioLegend, 306612), anti-human CD34–Alexa Fluor 700, (clone 561; BioLegend, 343622), anti-human CD7–APC (clone CD7-6B7; BioLegend, 343108), anti-human CD1a–PE (clone HI149; BioLegend, 300106), anti-human CD127–PE-Cy7 (clone A019D5; BioLegend, 351320), anti-human CD5–PE-Dazzle 594 (clone UCHt2; BioLegend, 300634), anti-human CD3–peridinin-chlorophyll-protein (clone SK7; BioLegend, 344814) and anti-human CD45-BV650 (clone HI30; BD Biosciences, 563717). The gating strategy was based on peripheral blood mononucleated cells; *n* = 2 biologically independent experiments. Cells were measured using a CytoFLEX S system (Beckmann Coulter); data were acquired using a CytExpert 2.5 software and analysed using the FlowJo v10 software.

### Patch clamp analysis of BG-HFO-derived CMs

BG-HFOs were individually dissociated into single cells using the STEMdiff cardiomyocyte dissociation kit and seeded (at a density of 2 × 10^3^ cells per well in RB+ containing 10 µM Y-27632) onto Geltrex-coated glass coverslips in a 12-well plate. The action potentials of BG-HFO-derived CMs were recorded by patch clamp technique in the whole-cell current clamp mode at room temperature. Cells were analysed 2–4 days after seeding using standard Tyrode solution in the recording chamber containing 140 mM NaCl, 5.4 mM KCl, 1.8 mM CaCl_2_, 1 mM MgCl_2_, 10 mM HEPES and 10 mM glucose adjusted to pH 7.4 with NaOH. The intracellular solution contained 120 mM k-gluconate, 10 mM Na-gluconate, 1 mM MgCl_2_, 10 mM HEPES, 10 mM EGTA/KOH and 3 mM Mg-ATP, adjusted to pH 7.2 with KOH. Borosilicate glass (1.2 mm outer diameter × 0.94 mm inner diameter; Harvard Apparatus, GC120TF-10) was used to pull micropipettes with resistances from 1.7 to 4.0 MΩ using a Sutter puller (Model P-97, Sutter Instruments), which were subsequently polished using a microforge (MF-900, Narishige). KCl (3 M) agar bridges set up the connection between the pipette solution and the amplifier. The liquid junction potential (16.2 mV) was calculated using the JPCalc software (P. Barry, University of South Wales, Sydney, Australia) and corrected a priori. Recordings were filtered at 5 kHz and sampled at 100 kHz using an Axopatch 200B amplifier and the Axon Digidata 1550 (Molecular Devices). The Clampfit 11.2 (Molecular Devices), Prism 7 (GraphPad Software) and CorelDRAW X8 (CorelDRAW Graphics Suite) software were used for data analysis and presentation.

### Calcium imaging of BG-HFOs

Selected BG-HFOs were used for calcium imaging, utilizing a Rhod-4 calcium assay kit. The material was prepared according to the manufacturer’s instructions. BG-HFOs were incubated with Rhod-4 dye-loading solution for 1 h in an incubator. The Rhod-4 dye-loading solution was then removed and the BG-HFOs were kept in RB+ for another 1 h in the incubator. Beats per minute counts of the organoids were performed using an Axio Observer A1 microscope, which was also used to take videos of beating BG-HFOs. Numerical source data used for the beats per minute-based statistical analyses are reported in Supplementary Table 1(iv).

### scRNA-seq

Two BG-HFOs were used for the single-cell analysis. The selected samples were individually washed in PBS w/o and dissociated with 100 µl Cardiomyocyte Dissociation Medium for 12 min at 37 °C. The organoids were individually washed with 3× the volume of RB+ medium, and the cells were counted and resuspended ensuring that there were 2 × 10^4^ cells per 65 µl PBS w/o with 0,04% BSA and 10 μM Y-27632 for each sample.

#### Library generation

Library preparation for scRNA-seq analysis of the mRNA was performed according to the Chromium NextGEM Single Cell 3ʹ Reagent Kits v3.1 user guide (manual part number CG000204 Rev B; 10x Genomics). According to the protocol, a given excess of cells was loaded to the 10x controller to reach a target number of around 1 × 10^4^ cells per sample. Fragment-length distribution of the generated libraries was monitored using a Bioanalyzer high sensitivity DNA assay (Agilent Technologies, 5067-4626). Quantification of the libraries was performed using a Qubit dsDNA HS assay kit (Thermo Fisher Scientific, Q32854).

#### Sequencing run

Generated mRNA expression libraries were pooled and sequenced on an Illumina NovaSeq6000 sequencer using one S4 (300 cycles) Flowcell by Novogene. Sequencing was performed according to the following settings: 150 base pairs as sequence reads 1 and 2, 8 base pairs as index read 1 and no index read 2. Two sequencing runs were conducted to reach a final coverage of around 4 × 10^4^ mean reads per cell.

#### Raw data processing

The 10x Genomics CellRanger analysis pipeline set (v7.0.0) was used with default parameters. Briefly, binary base call files were demultiplexed into FASTQ files by cellranger mkfastq using the respective sample sheet with utilized 10x barcodes. The cellranger count pipeline was used to align read data to the reference genome provided by 10x Genomics (human reference dataset refdata-gex-GRCh38-2020-A), counting aligned reads per gene with intronic reads included in the count matrix, and calculating the clustering and summary statistics. Outputs from the cellranger count of all samples were aggregated, normalized to the same sequencing depth and then the feature-barcode matrices were recomputed by cellranger aggr.

#### Data processing and analysis

The unique molecular identifier count matrices were imported to RStudio (v2022.07.1 Build 554; R v4.2.1) for further data processing and analysis. The R package SoupX (v1.6.2) (https://github.com/constantAmateur/SoupX), with default settings, was used to profile and remove ambient mRNA contamination^69^. The cleaned count matrices were read into the scRNA-seq workflow (https://github.com/ktrns/scrnaseq), a Seurat-based workflow, constructed by the Dresden-concept Genome Center (DcGC; https://genomecenter.tu-dresden.de)^70^. Gene annotations were retrieved from Ensembl hsapiens_gene_ensembl dataset (v98) (https://www.ensembl.org). The created Seurat objects were quality controlled and filtered for cells with 50–11,000 genes per cell and <30% mitochondrial reads per cell and genes expressed in more than five cells. The samples were merged, normalized running standard logarithm normalization and the normalized gene counts were then centred and scaled including counts and the percentage of mitochondrial gene as additional covariates to regress out. To determine the structure of the dataset with principal component analysis, 3,000 variable genes were selected. Seurat (v4.2.0) functions and Leiden algorithm (v0.4.3) were applied subsequently to construct a *K*-nearest-neighbour graph based on Euclidean distance in principal component space, to refine edge weights based on Jaccard similarity and to partition the graph. Data were visualized using UMAP projections. Specific cell clusters were identified using 13 principal components and a resolution value of 0.8. Marker genes that are differentially expressed in one cluster compared with all other clusters were identified based on raw RNA data and the method ‘MAST’. We require marker genes to be expressed in at least 25% of cells in the respective cluster, with a minimum log_2_-transformed fold change of one and adjusted *P* value of at most 0.05. The S4 class object was convert it to a SingleCellExperiment class object for cell-type annotation utilizing the SingleR package (v1.10.0) and the reference ‘BlueprintEncodeData’ datasets obtained from the celldex package (v1.6.0) for SingleR^71,72^. For visualization, pivotally functions of the packages Seurat and ggplot2 (v3.3.6) were employed. UMAP projections were imported to Loupe Cell Browser 6.0 (10x Genomics) to view annotated clusters and feature expression.

#### Mapping and annotating query datasets

We projected BG-HFO data as references onto HFO query data^28^ to annotate the cells of the query datasets by cell-type label transfer as described in the Seurat vignette ‘Mapping and annotating query datasets’ (https://satijalab.org/seurat/articles/integration_mapping.html; Seurat v5.0.2). As the datasets contain shared and unique cell types, the prediction scores for all cells (representing the strength of the prediction) were assessed and a prediction score threshold of 0.98 was used to exclude unlikely cell-type annotations.

#### CCC analysis

LIANA (v0.1.13; R implementation) was used to infer ligand–receptor interactions using multiple CCC inference methods (cellphonedb, logfc, sca, natmi and connectome) and the consensus resource as described in the LIANA tutorial (https://saezlab.github.io/liana/articles/liana_tutorial.html). LIANA combines the results of the used methods and calculates an aggregate_rank alongside an aggregated magnitude and specificity of each interaction. Interactions were filtered by aggregate_rank (≤0.01) and the frequencies of the inferred interactions for each pair of potentially communicating cell types were visualized^47^. Numerical source data obtained from the CCC analyses are reported in Supplementary Table 2. Gene-set enrichment analysis for cell-type-specific ligands and receptors obtained via LIANA was performed using Enrichr^48,49^ and the Gene Ontology Biological Process 2023 database. The obtained *P* values were computed from a Fisher’s exact test, which is a proportion test that assumes a binomial distribution and independence for probability of any gene belonging to any set.

### Statistics and reproducibility

HFOs and BG-HFOs as well as Cond.1, Cond.2 and Cond.3 organoids were cultured independently and are therefore considered independent biological units. Organoids (HFOs, BG-HFOs, Cond.1, Cond.2 and Cond.3) that showed the layered pattern of myocardial layer, inner core and outer layer were considered successfully formed and randomly chosen for downstream analyses. No statistical methods were used to pre-determine sample sizes but sizes are similar to those reported in previous publications^28,29^. Investigators were not blinded to the conditions of the experiments for data collection and analysis. For all following experiments, only successfully formed hES3 NKX2.e–eGFP-derived BG-HFOs, HFOs, Cond.1, Cond.2 and Cond.3 organoids (whereby HFOs were used as the control condition) were used; these organoids showed similar results in all experiments. Specifically, immunofluorescence staining (whole mount and cryosections) were repeated with at least three BG-HFOs from three independent experiments. Flow cytometry analyses, from which the statistics were derived, were repeated with between 3 and 19 independent groups of four or five pooled organoids (HFOs, BG-HFOs, Cond.1, Cond.2 or Cond.3)—whereby each group was considered as an independent biological unit—from up to 14 independent experiments. The precise numbers of repeats (*n*) are indicated in the respective figure legends. The CFU assays were performed *n* = 3 times for BG-HFO-derived CD34^+^, CD34^+^CD43^−^ and CD34^−^CD43^+^ cells using pooled BG-HFOs from three independent experiments, and *n* = 1 times for CD34^+^CD43^+^ cells. The lymphoid potential assay was performed *n* = 2 times using pooled BG-HFOs from two independent experiments. BG-HFOs derived from the iPSC line HSC_ADCF_SeV-iPS2 were analysed as follows: whole-mount immunofluorescence staining was repeated with at least three BG-HFOs from three independent experiments and flow cytometry analyses, from which the statistics were derived, were repeated with 8–10 independent groups of four or five pooled BG-HFOs—whereby each group was considered as an independent biological unit—from five independent experiments. Statistical analyses were performed using GraphPad Prism 7 and 9.1.2. For each experiment, outliers were statistically excluded using the tool ‘ROUT (Q = 10%)’ in GraphPad Prism. Statistical analyses were performed applying a two-tailed unpaired Student’s *t*-test assuming unequal variances for the comparison of two groups; for more than two groups, a one-way ANOVA with Bonferroni’s multiple comparison test or Sidak’s multiple comparison test was applied. The data met the assumptions of the statistical tests used, given that, when possible, normality (Shapiro–Wilk, D’Agostino and Pearson omnibus and/or Kolmogorov–Smirnov normality tests) was formally tested. The numerical sources used for the statistical analyses are reported in Supplementary Table 1. The data are presented as the mean ± s.e.m.; statistical significance was assigned as NS, not significant (*P* > 0.05), *P ≤ 0.05, **P ≤ 0.01, ***P ≤ 0.001 and ****P ≤ 0.0001.

### Reporting summary

Further information on research design is available in the Nature Portfolio Reporting Summary linked to this article.

## Online content

Any methods, additional references, Nature Portfolio reporting summaries, source data, extended data, supplementary information, acknowledgements, peer review information; details of author contributions and competing interests; and statements of data and code availability are available at 10.1038/s41556-024-01526-4.

## Supplementary information

Reporting Summary

Supplementary Video 1 Calcium imaging of a BG-HFO displaying synchronous contraction of the myocardial layer. The BG-HFO was stained with the calcium-sensitive dye Rhod-4 to enhance visualization of the contractions. Scale bar, 500 μm.

Supplementary Video 2 Calcium imaging of a BG-HFO displaying synchronous contraction of the myocardial layer. The BG-HFO was stained with the calcium-sensitive dye Rhod-4 to enhance visualization of the contractions. Scale bar, 500 μm.

Supplementary Video 3 Calcium imaging of a BG-HFO displaying circular beating of the myocardial layer. The BG-HFO was stained with the calcium-sensitive dye Rhod-4 to enhance visualization of the contractions. Scale bar, 500 μm

Supplementary Video 4 Fluorescence imaging of a BG-HFO displaying circular beating of the myocardial layer, observable via NKX2.5–eGFP transgene signal. Scale bar, 500 μm.

Supplementary Video 5 Calcium imaging of a BG-HFO displaying a wave-like contraction propagation from an initiation point. The BG-HFO was stained with the calcium-sensitive dye Rhod-4 to enhance visualization of the contractions. Scale bar, 500 μm.

Supplementary Video 6 Fluorescence imaging of a BG-HFO displaying a wave-like contraction propagation from an initiation point, observable via NKX2.5–eGFP transgene signal. Scale bar, 500 μm.

Supplementary Table 1 Workbook comprising all the numerical sources for the statistical analyses.

Supplementary Table 2 Dynamic Excel file including the numerical data obtained from the CCC analysis and used for the gene enrichment analysis.

## Data Availability

The gene expression datasets generated and analysed in the present study are available in the Gene Expression Omnibus repository (scRNA-seq raw data accession number GSE239748). Previously published gene expression datasets that were re-analysed here are available under the accession code GSE150202. All other data supporting the findings of this study are available within the article, its Supplementary Information and from the corresponding authors on reasonable request.
