# Supplementary material for: Blood-generating heart-forming organoids recapitulate co-development of the human haematopoietic system and the embryonic heart
Source: Nat Cell Biol. 2024 Oct 8;26(11):1984–96. doi: 10.1038/s41556-024-01526-4 (PMC11567889; doi:10.1038/s41556-024-01526-4)
Supplement: Supplementary file 1 — Reporting Summary [file 41556_2024_1526_MOESM1_ESM.pdf]

nature portfolio

Corresponding author(s): PD Dr. Robert Zweigerdt, Dr. Lika Drakhlis, Miriana Dardano

Last updated by author(s): Aug 8, 2024

Reporting Summary

Nature Portfolio wishes to improve the reproducibility of the work that we publish. This form provides structure for consistency and transparency in reporting. For further information on Nature Portfolio policies, see our Editorial Policies and the Editorial Policy Checklist.

Statistics

For all statistical analyses, confirm that the following items are present in the figure legend, table legend, main text, or Methods section.

n/a	Confirmed	
☐	☒	The exact sample size (*n*) for each experimental group/condition, given as a discrete number and unit of measurement
☐	☒	A statement on whether measurements were taken from distinct samples or whether the same sample was measured repeatedly
☐	☒	The statistical test(s) used AND whether they are one- or two-sided<br>*Only common tests should be described solely by name; describe more complex techniques in the Methods section.*
☒	☐	A description of all covariates tested
☐	☒	A description of any assumptions or corrections, such as tests of normality and adjustment for multiple comparisons
☐	☒	A full description of the statistical parameters including central tendency (e.g. means) or other basic estimates (e.g. regression coefficient) AND variation (e.g. standard deviation) or associated estimates of uncertainty (e.g. confidence intervals)
☐	☒	For null hypothesis testing, the test statistic (e.g. *F*, *t*, *r*) with confidence intervals, effect sizes, degrees of freedom and *P* value noted<br>*Give P values as exact values whenever suitable.*
☒	☐	For Bayesian analysis, information on the choice of priors and Markov chain Monte Carlo settings
☒	☐	For hierarchical and complex designs, identification of the appropriate level for tests and full reporting of outcomes
☒	☐	Estimates of effect sizes (e.g. Cohen's *d*, Pearson's *r*), indicating how they were calculated

Our web collection on statistics for biologists contains articles on many of the points above.

Software and code

Policy information about availability of computer code

Data collection	AxioVision Se64 Rel. 4.8<br>Zen 3.5<br>MACSQuant Analyzer 10 Software<br>CytExpert 2.5<br>10x Genomics CellRanger pipeline set v7.0.0<br>JPCalc software
Data analysis	AxioVision se64 Rel 4.8<br>Zen 2.6<br>FlowJo V10<br>GraphPad Prism 7 and 9.1.2<br>ImageJ 1.52p<br>Loup Cell Browser 6.0<br>10x Genomics CellRanger pipeline set v7.0.0<br>RStudio (v2022.07.1 Build 554; R v4.2.1)<br>R package SoupX (v1.6.2)<br>SingleR package (v1.10.0)<br>scrnaseq (DOI 10.5281/zenodo.7849063)<br>LIANA (v0.1.13; R implementation, https://saezlab.github.io/liana/articles/liana_tutorial.html)<br>CorelDRAW X8 (CorelDRAW Graphics Suite)<br>JPCalc software

March 2021

Clampfit 11.2 software

For manuscripts utilizing custom algorithms or software that are central to the research but not yet described in published literature, software must be made available to editors and reviewers. We strongly encourage code deposition in a community repository (e.g. GitHub). See the Nature Portfolio guidelines for submitting code & software for further information.

Data

Policy information about availability of data

All manuscripts must include a data availability statement. This statement should provide the following information, where applicable:

- Accession codes, unique identifiers, or web links for publicly available datasets
- A description of any restrictions on data availability
- For clinical datasets or third party data, please ensure that the statement adheres to our policy

The gene expression datasets generated and analyzed during the current study are available in the Gene Expression Omnibus repository: Single-cell RNA sequencing raw data have been deposited in Gene Expression Omnibus (GEO; accession number, GSE239748). Previously published gene expression datasets that were re-analysed here are available under accession code GSE150202. The reference 'BlueprintEncodeData' datasets was obtained from the celldex package (v 1.6.0) for SingleR. All additional data supporting the findings of this study are available within the article and its Supplementary Information.

Human research participants

Policy information about studies involving human research participants and Sex and Gender in Research.

Reporting on sex and gender	N/A
Population characteristics	N/A
Recruitment	N/A
Ethics oversight	N/A

Note that full information on the approval of the study protocol must also be provided in the manuscript.

Field-specific reporting

Please select the one below that is the best fit for your research. If you are not sure, read the appropriate sections before making your selection.

☒ Life sciences ☐ Behavioural & social sciences ☐ Ecological, evolutionary & environmental sciences

For a reference copy of the document with all sections, see nature.com/documents/nr-reporting-summary-flat.pdf

Life sciences study design

All studies must disclose on these points even when the disclosure is negative.

Sample size	No statistical methods were used to pre-determine sample sizes, but are similar to those reported in previous publications (Drakhlis et. al., 2021). For all the experiments containing statistical analyses a sample size of at least n = 3 up to 19 was chosen (precise n reported in corresponding figure legends), where each n consisted in a group of 4-5 successfully generated HFOs or BG-HFOs, identifyied as indeendent biological units, dissociated and pooled to homogenize the analyses. HFOs and BG-HFOs analyzed groups were generated from 3 up to 14 independent experiments (precise number reported in the corresponding figure legends).
Data exclusions	Not successfully formed HFOs and BG-HFOs were excluded (see "Replication"). For each experiment outliers were statistically excluded using the function "ROUT (Q = 10%) in GraphPad Prism
Replication	The HFO and BG-HFO that showed the typical NKX2.5-eGFP-layered pattern were considered successfully formed. All remaining HFOs and BG-HFOs (no proper layer formation) were considered failed. For each experiments 12-48 HFOs and/or 48-144 BG-HFOs were generated. For all following experiments, only successfully formed HFOs and BG-HFOs were used; the organoids showed reproducible results in all experiments.
Randomization	Randomization was not relevant to this study. Six different groups were analysed: HFOs from HES3 NKX2.5-eGFP, BG-HFOs from HES3 NKX2.5-eGFP, (Cond.1, .2, and .3) organoids from HES3 NKX2.5-eGFP and BG-HFOs from HSC_ADCF_SeV-iPS2103. For all the groups, all successfully formed organoids were used for further analyses.
Blinding	Data collection and analysis were not performed blind to the conditions of the experiments, since all data were analyzed using unbiased methods and blinding would not affect the results or the interpretation.

Reporting for specific materials, systems and methods

We require information from authors about some types of materials, experimental systems and methods used in many studies. Here, indicate whether each material, system or method listed is relevant to your study. If you are not sure if a list item applies to your research, read the appropriate section before selecting a response.

Materials & experimental systems

n/a	Involved in the study
☐	☒ Antibodies
☐	☒ Eukaryotic cell lines
☒	☐ Palaeontology and archaeology
☒	☐ Animals and other organisms
☒	☐ Clinical data
☒	☐ Dual use research of concern

Methods

n/a	Involved in the study
☒	☐ ChIP-seq
☐	☒ Flow cytometry
☒	☐ MRI-based neuroimaging

Antibodies

Antibodies used

anti-human CD31-APC (Miltenyi Biotec, 130-092-652)
anti-human CD31- PE (Miltenyi Biotec, 130-119-142)
anti-human CTNT-APC (Miltenyi Biotec, 130-106-711)
anti-human CD90-VIOBLUE (Miltenyi Biotec, 130-114-866)
anti-human CD34-VIOBLUE (Miltenyi Biotec, 130-124-459)
anti-human CD43-APCVIO770 (Miltenyi Biotec, 130-114-596)
anti-human CD45-PEVIO770 (Miltenyi Biotec, 130-110-634)
anti-human CD144-APC (Miltenyi Biotec, 130-125-985)
anti-human CD73-APC (Miltenyi Biotec, 130-112-061)
anti-human CD4-PEVIO670 (Miltenyi Biotec, 130-132-907)
anti human IgG1-PE (Miltenyi Biotec, 130-118-347)
anti-human IgG1-VIOBLUE (Miltenyi Biotec, 130-113-442)
anti-human IgG1-APCVIO770 (Miltenyi Biotec, 130-113-435)
anti-human IgG1-APC (Miltenyi Biotec, 130-113-434)
anti-human IgG1-PEVIO770 (Miltenyi Biotec, 130-113-440)
CD34 Microbead Kit UltraPure (Miltenyi Biotec, 130-100-453)
anti-human CD33-BV421 (clone P67.6; Biolegend, 366622)
anti-human CD14-BV421, (clone 63D3, Biolegend, 367144)
anti-human CD235ab-Pacific blue (clone HIR2, Biolegend, 306612)
anti-human CD34-AF700, (clone 561, Biolegend, 343622)
anti-human CD7-APC (clone CD7-6B7,24 Biolegend, 343108)
anti-human CD1a-PE (clone HI149, Biolegend, 300106)
anti-human CD127-PE-Cy7 (clone-A019D5, Biolegend, 351320)
anti-human CD5-PE-Dazzle 594 (clone UCHt2, Biolegend, 300634)
anti-human CD3-PerCP (clone SK7, Biolegend, 344814)
anti human CD45-BV650 (clone HI30, BD, 563717)
anti-CD31 (Agilent, JC70A)
anti-human CD31 (abcam, ab28364)
anti-human cTnT (abcam, ab64623)
anti-human cTnT (abcam, ab209813)
anti-human cTnT (Thermo Fisher Scientific, MA5-12960)
anti-human CD45 (abcam, ab8216)
anti-human NKX2.5 (Cell Signaling Technology, 8792)
anti-human vimentin (abcam ab92547)
anti-human CD144 (abcam, ab33168)
anti-human CD43 (abcam, ab101533)
anti-human RUNX1 (abcam, ab240639)
anti-human ALHD1A1 (Santa Cruz Biotechnology, sc-374149)
anti-human CD14 (abcam, ab182032)
donkey anti-rabbit Alexa Fluor 488 (Jackson ImmunoResearch, 711-545-152)
donkey anti-goat Alexa Fluor 488 (Jackson ImmunoResearch, 705-545-147)
donkey anti-goat Alexa Fluor 647 (Jackson ImmunoResearch, 705-605-147)
donkey anti-mouse Cy3 (Jackson ImmunoResearch, 715-165-150)
donkey anti-rabbit Alexa Fluor 647 (Jackson ImmunoResearch, 711-606-152)

Validation

All antibodies used were commercially validated by the respective manufacturer, the validations statements can be found on the manufacturer website, in the respective data sheets. Each lot of an antibody is tested following conformance, representative flow cytometry data and IF staining were always included in data sheets to demonstrate specificity and sensitivity to the relevant cell populations and types.
Moreover, for further validation of cTnT antibody (abcam, ab209813) on cryo-sections of BG-HFOs and cTnT antibody (Miltenyi) for FC, cardiac cells derived from directed cardiac differentiation were used, antibodies were used following manifacturer instructions.

For further validation of CD45 and CD43 antibodies (Miltenyi) for FC, hematopoietic cells derived from directed hematopoietic differentiation were used, following manifactuter instructions. For further validation of CD34, CD7, CD5, CD3 antibodies (Biolegend) and CD45 antibody (BD) for FC, peripheral blood mononucleated cells were isolated and used.

Eukaryotic cell lines

Policy information about cell lines and Sex and Gender in Research

Cell line source(s)	Human embryonic stem cell line:<br>HES3 NKX2.5-eGFP: Obtained from S. C. Den Hartogh and R. Passier (Department of Anatomy and Embriology, Leiden University Medical Centre).<br>Human induced pluripotent stem cell line:<br>HSC_ADCF_SeV-iPS2103: Obtained from A. Haase (Hannover Medical School)<br>Murine cell line:<br>MS5-hDLL4: MS5 murine stromal cells transduced with the third-generation lentiviral vector encoding human DLL4 (Delta like 4) and obtained from L. Lange (Hannover Medical School)
Authentication	All applied cell lines are authenticated and published:<br>HES3 NKX2.5-eGFP: Elliott et al., Nature Methods, 2011; Den Harthog et al., Stem Cells, 2015<br>HSC_ADCF_SeV-iPS2103: Haase et al., Stem Cell Research, 2017<br>MS5: Itoh et al., Experimental Hematology, 1989
Mycoplasma contamination	All cell lines were routinely tested for mycoplasma contamination and were tested negative.
Commonly misidentified lines<br>(See ICLAC register)	No such lines have been used.

Flow Cytometry

Plots

Confirm that:

- [x] The axis labels state the marker and fluorochrome used (e.g. CD4-FITC).
- [x] The axis scales are clearly visible. Include numbers along axes only for bottom left plot of group (a 'group' is an analysis of identical markers).
- [x] All plots are contour plots with outliers or pseudocolor plots.
- [x] A numerical value for number of cells or percentage (with statistics) is provided.

Methodology

Sample preparation	HFOs and BG-HFOs were individually dissociated into single cells using the STEMdiff Cardiomyocyte Dissociation Kit (Stemcell Technologies) and stained by applying the Fix&Perm kit (Nordic MUbio). For the erythro-myeloid and T-cell progenitor analyses, BG-HFOs were individually dissociated into single cells using the STEMdiff Cardiomyocyte Dissociation Kit (Stemcell Technologies). The obtained single cells were stained for the marker CD34 or for the markers CD34 and CD43; subsequently the cells were sorted. For the T-cell progenitor analyses, the generated ATOs were mechanically dissociated via pipetting. The obtained cells were strained through a 70 µm cell strainer to filter and exclude the murine stromal cells and were subsequently stained.
Instrument	Cells were measured at the MACSQuant Analyzer 10 (Miltenyi Biotec) or CytoFLEX S (Beckmann Coulter). Cells were sorted via Fluorescence Activated Cell Sorting (FACS) at the Dickinson FACSAria III Fusion (Biosciences) or via Magnetic Activated Cell Separation (MACS) using the OctoMACS separator (Miltenyi Biotec).
Software	Data were analyzed with FlowJo V10
Cell population abundance	Cells marked for CD34 and CD43 were sorted using Fluorescence Activated Cell Sorting (FACS) at the Dickinson FACSAria III Fusion (Biosciences). Cells marked for CD34 were sorted via Magnetic Activated Cell Separation (MACS) using an OctoMACS separator (Miltenyi Biotec).
Gating strategy	Living cells were gated within the FSC/SSC plot followed by doublet exclusion. Unstained samples were used to distinguish between negative and positive signals of antibodies. Isotype controls were used to validate the gating strategy. For the analyses of the T-cell progenitors, Fluorescence Minus One was used to compensate the panel and peripheral blood mononucleated cells were used as positive control for the gating.

- [x] Tick this box to confirm that a figure exemplifying the gating strategy is provided in the Supplementary Information.
